# Catechins and Their Therapeutic Benefits to Inflammatory Bowel Disease

**DOI:** 10.3390/molecules22030484

**Published:** 2017-03-19

**Authors:** Fei-Yan Fan, Li-Xuan Sang, Min Jiang

**Affiliations:** 1 Department of Gastroenterology, First Affiliated Hospital of China Medical University, 155 Nanjing North Street, Heping District, Shenyang 110001, China; feiyanfan7212@163.com; 2 Department of Geriatrics, First Affiliated Hospital of China Medical University, 155 Nanjing North Street, Heping District, Shenyang 110001, China

**Keywords:** catechins, inflammatory bowel disease, oxidative stress, mechanisms, tight junction functionality

## Abstract

Catechins are natural polyphenolic phytochemicals that exist in food and medicinal plants, such as tea, legume and rubiaceae. An increasing number of studies have associated the intake of catechins-rich foods with the prevention and treatment of chronic diseases in humans, such as inflammatory bowel disease (IBD). Some studies have demonstrated that catechins could significantly inhibit the excessive oxidative stress through direct or indirect antioxidant effects and promote the activation of the antioxidative substances such as glutathione peroxidases (GPO) and glutathione (GSH), reducing the oxidative damages to the colon. In addition, catechins can also regulate the infiltration and proliferation of immune related-cells, such as neutrophils, colonic epithelial cells, macrophages, and T lymphocytes, helping reduce the inflammatory relations and provide benefits to IBD. Perhaps catechins can further inhibit the deterioration of intestinal lesions through regulating the cell gap junctions. Furthermore, catechins can exert their significant anti-inflammatory properties by regulating the activation or deactivation of inflammation-related oxidative stress-related cell signaling pathways, such as nuclear factor-kappa B (NF-κB), mitogen activated protein kinases (MAPKs), transcription factor nuclear factor (erythroid-derived 2)-like 2 (Nrf2), signal transducer and the activator of transcription 1/3 (STAT1/3) pathways. Finally, catechins can also stabilize the structure of the gastrointestinal micro-ecological environment via promoting the proliferation of beneficial intestinal bacteria and regulating the balance of intestinal flora, so as to relieve the IBD. Furthermore, catechins may regulate the tight junctions (TJ) in the epithelium. This paper elaborates the currently known possible molecular mechanisms of catechins in favor of IBD.

## 1. Introduction

### 1.1. Inflammatory Bowel Disease (IBD)

IBD, which contains two major manifestations, namely ulcerative colitis (UC) and Crohn’s disease (CD) [1], refers to idiopathic gut chronic inflammatory disease. It is characterized by the remission and exacerbation of clinical syndromes which are characterized by intestinal bleeding and diarrhea, leading to the disruption of the epithelial barrier and the formation of epithelial ulceration [2], with extensive areas of structural damage to the intestine [3]. The clinical manifestations of IBD that depend on the intestinal segment involved generally include haematochezia, diarrhea, abdominal pain, passage of pus, mucus, blood, obstruction, fever, weight loss, wasting, and ultimate development into cancer [4,5]. In addition, more than 25% of IBD patients are also suffering from extra-intestinal complications, including peripheral arthritis, axial arthropathies, erythema nodosum, pyoderma gangrenosum, episcleritis, iridocyclitis, anemia, etc. [3,4].

With the rise of its incidence and prevalence in Eastern Europe and Asia, especially in western countries (being as high as 1%), IBD has gradually become more prevalent worldwide [5]. Even today, its aetiology and pathogenesis have not been completely elucidated. However, it has been made clear that IBD is a complex multifactorial disease which involves the interaction between the host gene component, the intestinal immune system, environmental factors, the “in-vironment”, and the complex intestine microbiome [6]. Enormous molecular- and genome-wide-related studies have revealed the existence of distinct changes in the gut microbiota of IBD and also have elucidated the importance of “dysbiosis” of the gut microbiome in the aetiopathogenesis of IBD [6]. For example, the invading pathogens and the enteric commensal bacteria which have been transformed from a symbiotic to a putative pathogenic one, or its production, can initiate the immune response of IBD in genetically-susceptible individuals [6]. After reacting with the antigens, such as formyl-Met-Leu-Phe (fMLP) and lipopolysaccharide (LPS), the intestinal epithelia or the macrophages, neutrophil cells will be stimulated [7,8]. Then, the activated cells can infiltrate, proliferate, differentiate, and produce different kinds of inflammatory cytokines and chemokines, pro-inflammatory cytokines, and reactive oxygen species (ROS), thus initiating and expanding the inflammation, while resulting in widespread lesions to cells and tissues [3,9,10]. On the contrary, there are also meaningful studies which have demonstrated that the neutrophils and macrophages would play a protective role in colitis by secreting immunosuppressive factors or other factors [11,12,13].

IBD is characterized by excessive Th1 or Th2 cell responses, which have, respectively, become more common in CD or UC [14]. Th1 cells, the chief mediators in type 1 immunity, can secrete interferon-γ (IFN-γ) (the iconic product of Th1 cells), interleukin 12 (IL-12), and tumor necrosis factor-α (TNF-α) which, in turn, induce TNF-α, IL-6, and IL-1β to release from macrophages. Wang et al. have found that the clinical course of CD-like mouse colitis has been improved by inhibiting the activation of Th1/Th17 cells [15]. The activated Th2 cells and the cytokines secreted by Th2 cells, such as IL-4, IL-5, IL-9, IL-10, IL-23, and IL-25, have increased in inflamed mucosa of UC patients than in CD ones [3]. In a distinct activation pathway from Th1 and Th2 cells, the increased number of activated Th17 cells and secreted cytokines, such as IL-17A and IL-17F, in IBD patients and animals in studies have suggested that Th17 cells play an important role in the development of IBD [16,17]. Additionally, many authors have reported that T-regulatory (Treg) cells, characterized by the expression of fork-head box P3 (FOXP3), IL-10, and TGFβ1, play a fundamental role in maintaining the gut immune-regulatory properties and inhibiting the activation of Th1, Th2, and Th17 cells [16,18,19].

**Figure 1:**
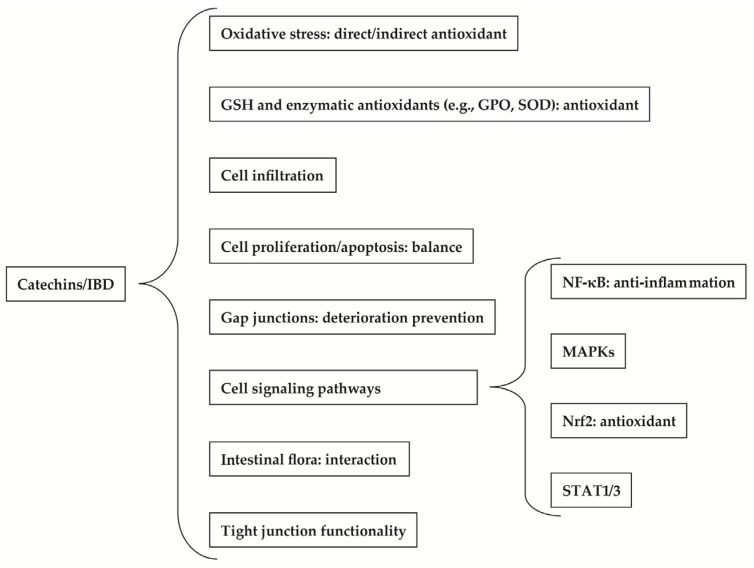
The main targets of action of catechins in IBD according to this article.

The main problems faced by the current therapeutic strategies for IBD include limited benefits, numerous side effects, and the weak responsiveness to patients who take anti-inflammatory drugs [20,21,22,23]. A population-based study has indicated that the widely-used first-line therapies, corticosteroids (CSs), can merely help a part of IBD patients who needed CSs to achieve remission, compared with 16% who failed to respond to CSs. However, over time, a prolonged response or steroid dependence will appear [24]. These motivated the imminent requirement of alternative treatment with high efficacy but less toxic side effects. Dietary polyphenols have been considered as novel therapies [5,25]. Only a few human studies, which made use of pure polyphenol or natural plant extracts, have demonstrated the effects of plant polyphenols, such as curcumin and pycnogenol, on IBD [14,26,27]. A randomized, double-blind, multicenter human trial indicated that curcumin seemed to be a promising and safe choice for keeping remission in patients with quiescent UC [27]. Pycnogenol has been proved to significantly reduce the markers of oxidative stress, but not markers of inflammation, indicating the positive effects of mid-long-term administration of hydrophilic polyphenols in CD patients [26]. Further human trials employing polyphenols are needed to confirm the beneficial effects of polyphenols in the management of IBD. At the same time, catechins, including some kinds of polyphenols, have also gained increasing attention over the past few years [8,9,14,28]. Owing to their potential for anti-inflammation, anti-oxidation, and anti-bacterial activities, they contribute to the remission of IBD. A human study about the effects of polyphenon E (a standardized green tea preparation consisting of 65% epigallocatechin gallate (EGCG) and 22% other catechins) on IBD showed the improvement in response and in the remission rate in mild to moderate, but refractory, UC patients [14]. How these catechins express their power is the focus of this review (Figure 1).

### 1.2. Overview of Catechins Activity

**Figure 2:**
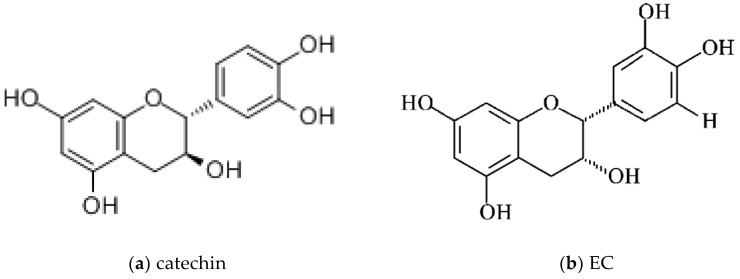

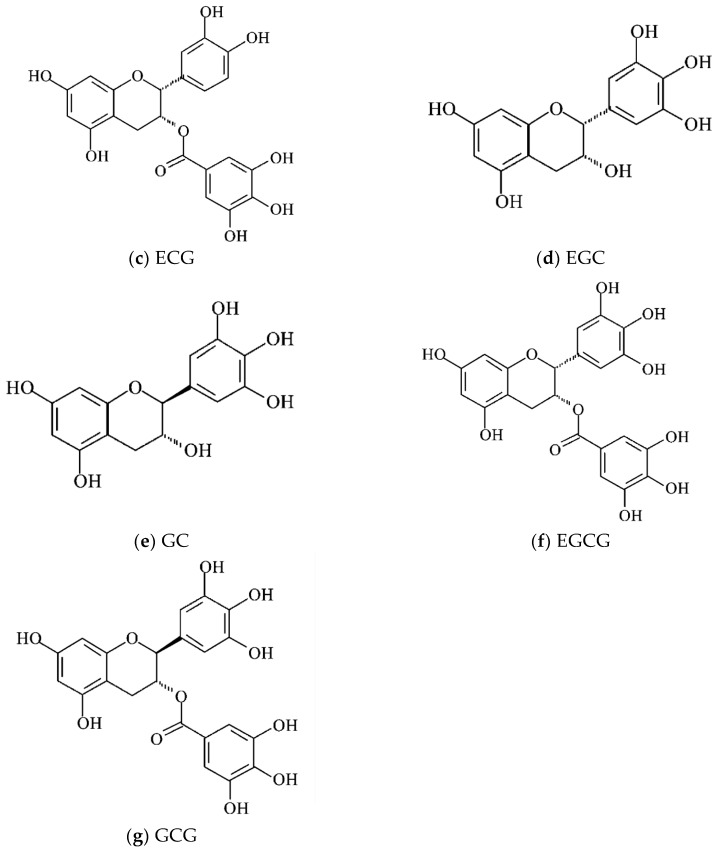
The chemical structures of major kinds of catechins. (**a**) catechin: (2*R*,3*S*)-(+)-catechin; (2*R*,3*S*)-2-(3,4-dihydroxyphenyl)-3,4-dihydro-2*H*-chromene-3,5,7-triol; (**b**) EC: (2*R*,3*S*)-(–)-epicatechin; 2-(3,4-Dihydroxyphenyl)-3,4-dihydro-2*H*-1-benzopyran-3,5,7-triol; (**c**) ECG: (2*R*,3*R*)-(–)-epicatechin gallate; (2*R*,3*R*)-2-(3,4-dihydroxyphenyl)-3,4-dihydro-1(2*H*)-benzopyran-3,5,7-triol 3-(3,4,5-trihydroxybenzoate); (**d**) EGC: (2*R*,3*R*)-(–)-epigallocatechin; (2*R*,3*R*)-2-(3,4,5-trihydroxyphenyl)-3,4-dihydro-1(2*H*)-benzopyran-3,5,7-triol; (**e**) GC: (2*S*,3*R*-(–)-gallocatechin; (2*S*,3*R*)-2-(3,4,5-trihydroxyphenyl)-3,4-dihydro-1(2*H*)-benzopyran-3,5,7-triol; (**f**) EGCG: (2*R*,3*R*)-(–)-epigallocatechin gallate; (2*R*,3*R*)-2-(3,4,5-trihydroxyphenyl)-3,4-dihydro-1(2*H*)-benzopyran-3,5,7-triol 3-(3,4,5-trihydroxybenzoate); (**g**) GCG: (2*S*,3*R*)-(–)-gallocatechin gallate; (2*S*,3*R*)-2-(3,4,5-trihydroxyphenyl)-3,4-dihydro-1(2*H*)-benzopyran-3,5,7-triol 3-(3,4,5-trihydroxybenzoate).

Catechins are known as a type of polyphenols naturally occurring in certain foods and medicinal plants, such as legumes, *Rubiaceae*, stem bark of the *fabaceae* species, including the Mimosoideae subfamily, such as *Abarema cochliacarpos*, teas (green tea, pu-erh tea, pu-erh green tea), *Mouriri pusa* Gardn, buckwheat, grapes, cocoa beans, litchis, and apples [29,30,31,32,33,34,35,36]. Catechins have been regarded as the characteristic compounds in green tea for daily beverage and crude medicine in Asia, especially in China and Japan, for thousands of years. In general, catechins mainly include catechin, epicatechin (EC), epicatechin gallate (ECG), epigallocatechin (EGC) and its stereoisomer gallocatechin (GC), EGCG and its stereoisomer gallocatechin gallate (GCG), with their compositions being similar for each other [36,37,38,39] (Figure 2). Catechins are the most active constituent polyphenols in the green tea (*Camelia sinensis*) [3]. EGCG, which accounts for almost 50% of the total catechins content of green tea extracts, makes up to 30% of the dry weight of green tea leaves [40], and has the strongest chemopreventive potential containing anti-inflammatory, anti-mutagenic, and anti-carcinogenic effects among the green tea catechins which contain EC, EGC, and EGCG [3,41,42]. Over the past few years, catechins have attracted interest due to their presumed roles in various physiological accommodative activities, such as anti-hypertensive [9], antibacterial [36], anti-inflammatory [29,43], and antioxidative activities [34,40,44,45], and as a protective effect from atherosclerosis. Moreover, increasing numbers of studies have demonstrated that catechins could also possess the anti-carcinogenic activity in many experimental systems and in many kinds of organs, including the oral cavity, esophagus, stomach, small intestine, colon, lung, liver, pancreas, skin, prostate, mammary gland, and bladder [46,47,48,49,50]. It has been proved that catechins could inhibit carcinogenesis, tumor growth, cancer cell invasion, and tumor angiogenesis, by suppressing the induction of proangiogenic factors, such as vascular endothelial growth factor (VEGF) [51]. It was established that EGCG, relying on its very strong reducing property, can suppress the activity of urokinase, an enzyme used by tumor cells to invade and metastasize, and the catechins can be safer than the synthetic inhibitor of urokinase activity [52].

At the same time, some researchers have focused on the fact that catechins have been proven to be effective in anti-tumor activity and for the suppression of inflammation which results in cell injury and death, contributing to many diseases, such as IBD, lung injury, and inflammation-induced carcinogenesis in various kinds of models [14,41,53]. Thus, catechins may be effective therapies to relieve IBD without obvious side effects in various cell, animal, and human experiments [53], which may concentrate on the catechin, EC, ECG, EGCG, or plant extracts. Studies have showed that the dietary polyphenol levels of 0.12% and 0.24% decreased the azoxymethane (AOM)-induced aberrant crypt foci (ACF) formation in a rat model [49], which was also observed with EGCG (0.01% and 0.1% in drinking water) in rats, too [54]. It was described that EGCG applied intraperitoneally at 50 mg/kg body weight was shown to inhibit colon inflammation in colitis in rat models, resulting in the decrease of weight loss and shortening in the length of the colon [40]. In another study by Vasconcelos et al., EC (10 mg/kg) has also been approved to benefit colitis induced by 2,4,6-trinitrobenzenesulfonic acid (TNBS) in acute and chronic rat models [30]. In Caco2 cells, ECG has shown comparable antioxidant capacities with a dose response between 10 and 100 μM [55]. However, the beneficial effects of catechins on the intestinal lesions have disappeared in higher doses in previous studies. For example, dietary green tea polyphenols (GTP, including EC, EGC, ECG and EGCG. Among them, greater than 40% is EGCG [56]), at dose levels of 0.5% and 1%, profoundly enhanced the dextran sodium sulfate (DSS)-induced acute colitis, but the 0.1% and 0.25% levels of GTP in the diet had a tendency to decrease in 1,2-dimethylhydrazine (DMH)-induced male ICR mice [57]. A high dose of EGCG was also demonstrated to act as a prooxidant [57,58], instead of antioxidant [59], by generating ROS and even causing damage to cellular DNA in various kinds of cells. Guan et al. suggested that 0.5% dietary EGCG could exacerbate DSS-induced colitis in inflamed colons of mice models as compared with those on the AIN93M diet [50]. In addition, the dose of 0.3% EGCG in the rodent diet corresponds to a daily ingestion of 1.5 g EGCG for individuals with daily requirements of 2000 kcal [50]. However, the detailed mechanisms by which EGCG promotes inflammation in animal models are not clear yet, and it is probably related to the antiplatelet and antithrombotic activities of EGCG [60]. This phenomenon could also be observed in TNBS-induced rats’ colitis, so that the anti-inflammatory effect of 10 mg/kg dose of EC will disappear with higher doses [30]. A study in vitro of human neutrophils showed that EGCG concentrations ranging from 0 to 30 µM were not toxic to the cells. However, when treated with 100 and 400 µM of EGCG, cell membrane integrity decreased [61]. Additionally, 10 µM of EGC could cause an obvious loss of cell membrane integrity, which will happen in ECG levels of 3 and 10 µM, too. EC also caused cell death at a concentration of 2 µM or more [61]. However, 30 µM of EGCG, 3 µM of EGC, 2 µM of ECG, and 1.4 µM of EC, either alone or in combination, present marked immunomodulatory actions by regulation of inflammatory cytokines, reduction of ROS production, and migration of neutrophils [61], and relevant evidence has indicated that the pro-inflammatory capacity may exceed the benefits on gut inflammation in the application of high concentration of catechins [30]. Thus, the concentrations of catechins play an important role when they are applied to intestinal inflammation. However, most of today’s trials for catechins in IBD are related to the anti-inflammation capacity. On the other hand, due to various reasons, like the lack of specific observational and epidemiological animals and human studies aiming to define the quantitative effective concentrations, as well as due to the fact that the materials for studies are always a mixture extract of various kinds of plants, it is still unable to provide exact, or even equivalent, effective dose ranges of catechins in vivo and in vitro in detail. Nowadays suggested dosages of various marketed supplements often have no scientific basis [62]. A pilot study suggested that the oral concentration of EGCG, up to 400 mg twice daily, achieved the immunocompetence effect in UC patients [14]. However, the upper limit of the dose of EGCG is not still fully indicated [2,14]. The catechins, and the corresponding plants’ optimal doses, need to be further defined; for example, Kim et al. demonstrated that the daily oral intake of 6 g of tea would induce a corresponding adverse reaction [57], with three cups of tea generally containing about 500 mg of green tea [40].

#### 1.2.1. Catechins: Chemical Structures

Catechins are composed of types of plant polyphenols, which ubiquitously exist in foods and medical plants, such as green tea. In terms of chemical structures, polyphenols are compounds which have different amounts of phenolic rings attached by two or more hydroxyl groups [3], resulting in more than 8000 dietary polyphenols [44], which have thousands of chemical structures, varying from simple molecules to highly polymerized compounds [10]. Acting as hydrogen or electron donors, the common phenol groups can scavenge free radicals [3]. Among them, flavonoids link two aromatic rings (A and B) via a three-carbon chain forming an oxygenated heterocycle (C ring), ending in a common C6-C3-C6 structure, by reacting with different groups of hydroxyl and glycosidic groups and binding with other molecules [10,44]. Flavonoids are divided into various subfamilies. Flavanols are one kind of the subfamily which has a hydroxyl in position 4 attached to a saturated C ring. Catechin, and its stereoisomers in *cis* or *trans* configuration, with respect to carbons 2 and 3, ((–)-EC (*cis*) (6.4% approximately of total green tea catechins) and (+)-catechin (*trans*)) are flavan-3-ol compounds [10,44] (Figure 2). Through esterification with gallate groups, flavanols can form EGCG, the gallic acid ester of EGC at position 3, approximately half of total green tea catechins [63], and ECG (approximately 13.6% of total green tea catechins) [9] (Figure 2).The catechins mainly comprise catechin, EC, ECG, EGC, and EGCG [9,10]. Monomeric catechins can further react and synthesize the condensed catechins which are produced by random polymerization [10,44,64]. Then, the chemical structures of the resulting dimeric condensed catechins are defined by not only the monomeric catechins, but also the pathway of link among monomers [44]. For example, the most common oligomers derived from EC (i.e., procyanidins) are classified into A-type and B-type, depending on the plant, respectively, and generally present in peanuts and coca (*Theobroma cacao*). Chemically, in A-type dimers, the monomers are linked by both a 4 → 8 carbon–carbon and a 2 → O7 ether bond, and the monomers of the B-type dimers are linked through 4 → 8 carbon–carbon bonds [10]. The isomerization, the arrangement of groups adhered to the aromatic ring, monomer bonding styles, and levels all have an important impact on the biochemical function. Moreover, catechins will decompose or produce secondary compounds, like the formation of glucuronide, sulfate, and methyl metabolites, acetylate conjugates and quinone type metabolites, and even microbial metabolites including phenolic acids and their glycine conjugates etc. [10,39,40,65,66,67]. The production of condensation reactions of catechins, or their derivatives, will have an effect on their biological effects [40]. For example, the intracellular EGCG-3′′-glucuronide can be decreased by piperine, resulting in higher free EGCG [40]. Thus, maintaining the stability of the chemical structure is essential to the biochemical functions of catechins.

#### 1.2.2. The Bioavailability of Catechins

**Figure 3:**
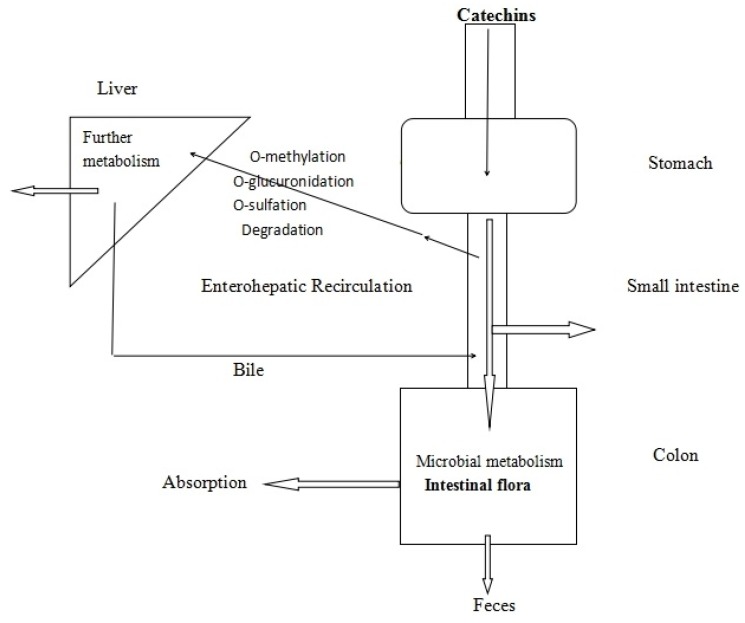
Supposed metabolism of green tea catechins in humans.

Catechins can be absorbed by the gastrointestinal tract (GIT), as catechins themselves, and their metabolites, are (mainly) formed in the acidic environment of the stomach, enterohepatic recirculation, the mucosa of the small intestine, and the microbial metabolites in the colon [35,66] (Figure 3). All catechins may undergo three types of metabolic pathways: methylation, glucuronidation, and sulfation in the liver and intestinal tissues in humans [67], like (epi)catechin transfering into plasma itself, or the corresponding glucuronide, sulfate, and methylated metabolites produced by uridine-5′-diphosphate glucuronytransferase, sulfotransferase, and cate-chol-*O*-methyltransferase enzymes [66]. Stalmach et al. have revealed that some flavan-3-ol monomers are absorbed in the upper part of the GIT in a human study [68]. Then, most of the ingested flavan-3-ols and their metabolites, formed in the upper GIT and transported back into the intestinal lumen, will reach the large intestine. On the deglucosidation function of mammalian β-hydrolases like lactase-phlorizin hydrolase (LPH) in the intestinal epithelial cells, and some hydrolases from the intestinal microflora like glucuronidase, catechins, and types of flavonoids, will finally form the corresponding aglycones, then be absorbed into human circulation and undergo further transformation and metabolism, which is believed to be the first and important step in the absorption of flavonoids [69,70]. Under the function of gut microbiota, they will also turn into phenolic acids and lactone derivatives, which will be absorbed or pass in the feces [65]. The microbial metabolites and their hepatic conjugates are distributed to tissues and are excreted by urine [71]. Investigations in humans have underlined that more of the flavan-3-ol metabolites were derived from gut flora-associated catabolites coming from the colon rather than from the upper GIT in the body [66]. As shown in a human study, colonic flora-derived flavan-3-ol catabolites (polyhydroxyphenyl-γ-valerolactones) were the main substance in urine, with higher concentrations than flavan-3-ol conjugates [72].

In the plasma following the administration of decaffeinated green tea (DGT) or pure catechins, more than 80% of green tea catechins were found to exist in their conjugate forms [67]. Similarly, EGC was mostly in the glucuronide form (57%–71%), the sulfate form (23%–36%), and the free form (3%–13%). After oral administration, the urinary excretion levels of four catechins: (+)-catechin, ECG, EGC, and EGCG, ranged from 0% to 9.8%, except for (+)-catechin with 23.6%–28.2% in human volunteers [67]. Urinary excretion of metabolites during a 24 h period after green tea consumption corresponded to 28.5% of the ingested (epi)catechin and 11.4% of (epi)gallocatechin. Human study has revealed that after absorption in the small intestine, the peak plasma concentrations (nmol/L) of (epi)catechin and (epi)gallocatechin glucuronide and their conjugated metabolites would be reached, varying from 1.6 to 2.3 h [66]. Then, the plasma concentration rapidly decreases due to the short apparent half-life, while few dietary catechins and related phenolic compounds are not changed, except for ECG and EGCG in plasma [66].

The bioavailability of catechins may be influenced by flavan-3-ol stereochemistry, enterohepatic recirculation, and the impact of dose and other food components on it [35,40,66,67,71]. Ottaviani et al. investigated in humans the administration of equal quantities of (–)-EC, (–)-catechin, (+)-EC, and (+)-catechin. The bioavailability of these stereoisomers were ranked as (–)-EC > (+)-EC = (+)-catechin > (–)-catechin on the basis of plasma concentrations and urinary excretion of the aglycones [73]. Studies demonstrated that enterohepatic recycling could improve the bioavailability of polyphenols. Hepatic metabolites will be excreted in the bile and enter the small intestine [74], then after the enzymes, like glucuronidase, coming from gut microbes, the formed aglycones of catechins can be reabsorbed again [35]. The bioavailability can be influenced by the dose of catechins. In ileostomists, 0–24 h urinary excretion of the (epi)gallocatechin metabolites did not increase significantly with intakes of 22 μmol, 55 μmol, and 165 μmol. However, urinary excretion increased significantly to 107 μmol and 262 μmol from 36 μmol, when the ingestion of 77 μmol (epi)catechins are increased to doses of 192 μmol and 577 μmol [66]. When combined with different polyphenols, catechins may show very different bioaccessibilities, too. Owing to the existence of material, such as sucrose and ascorbic acid which can maintain stability and protect from degradation in alkaline conditions, the bioaccessibility of catechins has been up-regulated. This may explain that, in rats, chocolate including higher sucrose levels enhanced the plasma concentration of catechins metabolites than milk and dark chocolates [75]. It has been found that ECG, EGCG, and some metabolites of EGCG can strongly inhibit the methylation of EGC [67].

As we can see, after GIT application, catechins show a low absorption and bioavailability, leading to a reduction of the effect on the tissues. However, most of catechins and their metabolites reach high concentrations in the GIT [3]. Therefore, some suppose that the potential benefit from catechins would be more useful for gastrointestinal disease [2,40,55].

## 2. Therapeutic Benefits of Catechins to IBD

It has not yet been entirely made clear that the mechanisms by which catechins benefit inflammatory diseases, such as IBD, while human experiments demonstrating that catechins can improve IBD are still scarce so far. However, there is abundant evidence showing that catechins may work through a combination of oxidation inhibition, alteration of cellular signaling, and regulation of intestinal flora [29,53,76].

### 2.1. Effect of Catechins on Oxidative Stress

The imbalance between the oxidative reactions and antioxidative defenses plays an important role in the pathogenesis of IBD [3,30,77]. IBD is considered as one of the major oxyradical-overload diseases, with a cancer-prone phenotype [7]. Kruidenier et al. have also shown the excessive production of ROS and radical nitrogen metabolites can be observed in the gut of IBD patients [78]. In general, oxidant species are referred to as ROS [10], including oxygen as a superoxide anion (O_2_^−^), hydroxyl radical (OH·), and singlet oxygen (^1^O_2_) [36,40]. ROS activation is essential in the normal regulation of cell signaling [79,80]. When different ROS overwhelm the antioxidant defenses of tissue, they likely contribute to the functional disruption of the intestinal mucosa [3], by causing damage to cellular lipids, proteins, cytoskeleton, even DNA, and ultimately increase the gut permeability, then destroy the gastrointestinal barrier integrity [40]. Furthermore, the imbalance can stimulate an inflammatory cascade by enhancing the expression of signaling pathways, such as nuclear factor-kappa B (NF-κB) and mitogen-activated protein kinases (MAPKs), enlarging the inflammatory events, even colon cancers, in accordance with the notion of Brückner et al. [3,29,40].

Najafzadeh et al. have also proved that in vitro lymphocytes from IBD patients, when treated by EC, showed a protective effect against oxidative stress induced by 2-amino-3-methylimidazo[4,5-*f*]-quinolone (IQ), reliably protecting cells from the damaging effects of ROS in IBD where levels of ROS are highly increased [81]. Renato et al have shown that (+)-catechin has the antioxidant capacity in a dose-dependent way between 0 and 100 μM [82]. The study of antioxidant effects of catechins has shown that EGCG, ECG, and peracetylated (–)-epigallocatechin-3-gallate (AcEGCG) can significantly lower the ROS levels induced by LPS in RAW264.7 cells [58]. It has also been proposed that the high doses of EGCG can produce ROS to be a prooxidant, inducing the activation of the transcription factor nuclear factor (erythroid-derived 2)-like 2 (Nrf2) pathway [58].

As antioxidative agents, catechins possess both “direct antioxidant effects” and “indirect antioxidant effects”. The catechins can scavenge free radicals and chelate redox-active metals, making them direct antioxidants. At the same time, as indirect antioxidants, catechins regulate protein synthesis activities and signaling strategies, such as mediating the property of prooxidant enzymes. Both capacities are dependent on concentrations, respectively, in high or low dose, in which the high dose can demonstrate more adequate antioxidant capacity in the digestive tract [10,44,62].

**Figure 4:**
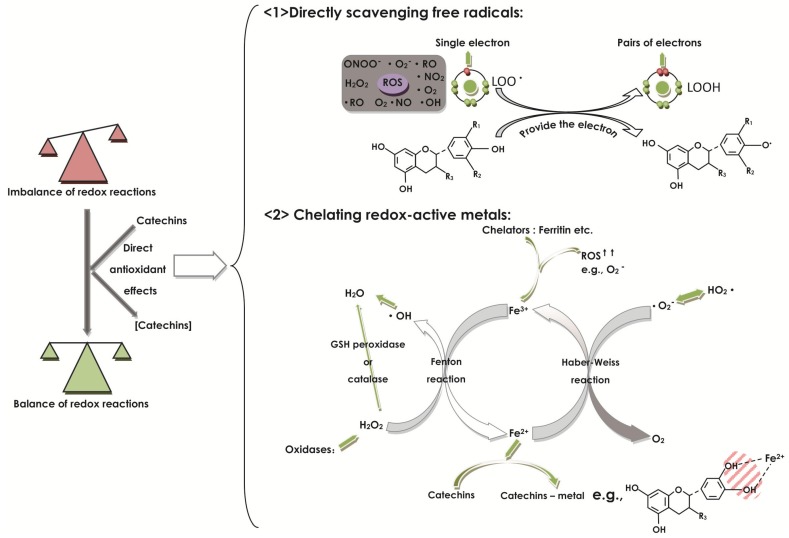
Catechins can exert direct antioxidant effects by reacting with free radicals to disrupt the free radical chain reaction and chelating redox-active metals.

Catechins can exert direct antioxidant effects with free radical scavenging and redox-active metal sequestration in the digestive tract (Figure 4). Catechins have different types of diastereoisomers like EC, ECG, EGC, and EGCG, but they all compose a similar chemical structure to stabilize the free radicals, which are related to the phenolic groups [43,44]. Additionally, catechins are successful in achieving high concentrations in the digestive tract [10]. Catechins, as kinds of polyphenols, have the common characteristic of free radical scavengers because of the common chemical structure of phenolic hydroxyl groups in polyphenols [44], in which catechin has shown the most powerful capacity of freeing the radical scavenger [36]. A study to assess the effect of (+)-catechin on the LPS-induced murine peritoneal macrophages suggested that (+)-catechin is capable of significantly inhibiting the intracellular ROS by removing free radicals and NO production, showing antioxidant ability, confirming that property against the ROS and reactive nitrogen species (RNS) activity in RAW264.7 macrophages or in vitro [29].

As free radical scavengers (Figure 4), catechins are able to react with free radicals to disrupt free radical chain reactions, like the common oxidative reaction of lipid oxidation. The exact mechanisms shared by polyphenols are (i) catechins can donate a one-electron of phenolic OH groups to reduce free radicals (for example: LOO· + EC = LOOH + EC·) [44]; (ii) the aromatic group can maintain stability through the resonance of the resultant aroxyl radicals [83]. For example, the free radical (LOO·) with a higher E°’ will get the electron of the catechins with a lower E°’, resulting in breaking the free radical chain reaction (e.g., LOO· + POH = LOOH + PO·). The potency of the parent polyphenol to disrupt the free radical chain reaction is dependent on the stability of the produced PO·. The more stable the PO· is, the greater potency the parent polyphenol has [44]. In addition, there is no convincing experiment to compare the reducibility between catechins that show extensive antioxidant activity in vitro and GSH, which belongs to an endogenous antioxidant system and, generally, is in both reduced (GSH) and oxidized (GSSG) forms. Whereas, when pretreated with Aβ, the GSH content is reduced, but after addition of EGCG, the content of GSH will be restored again in BV2 cells [84], indicating that the reduction of EGCG is greater than that of GSH. However, generally, catechins increase the activity of GSH by forming types of complexes with greater reduction in the B ring. However, it is needed to compare the reducibility between catechins and GSH by using further more precise and specific experiments, like applying chromogenic compounds to assess the ability of the antioxidant to trap free radicals [82]. As another mechanism of direct antioxidant effect of high doses of catechins, catechins have the common structure to chelate redox-active metals (Figure 4). By reacting with (free or poorly liganded) Fe^2+^ in the Fenton reaction, hydrogen peroxide (H_2_O_2_) can produce Fe^3+^, and by reacting with Fe^3+^ in the Haber–Weiss reaction, superoxide produces Fe^2+^, thereby resulting in redox cycling. When occurring in pathological changes, the imbalance of iron and ROS like over-production of ROS may free iron from chelators, like ferritin, producing more ROS [85,86]. As a type of flavonoids, catechins can bind iron, like in flavonoid-metal chelation, which occurs preferentially at the 3-hydroxyl-4-carbonyl group, firstly, then the 4-carbonyl-5-hydroxyl group, and the 3′-4′ hydroxyl (if present) [87], thus inhibiting the activity of redox-sensitive metal and decreasing the production reaction of free radicals [44]. Finally, the basic form of this kind of antioxidant effect depends on the E°’ of each polyphenol-metal complex. For being an antioxidant, the polyphenol-metal complex should have a lower power in radical formation, as compared with the physiological metal-complexes [44].

Catechins in low concentrations have indirect antioxidant capability for inhibiting the modulation of enzymes that generate oxidants, like NADPH-oxidase (NOX), and change the oxidant production by inhibiting the combination of ligands with receptors, such as TNF-α to TNF-αR, in gut cells [10]. Biasi et al. have shown that EGCG could inhibit NOX and the following enhanced ROS [3]. It has been proposed that EC and its metabolites could inhibit NOX, resulting in the decrease of superoxide production. One of EC metabolites, *O*-metabolite, has a similar structure to apocynin, a classical NOX inhibitor [10,44]. EC may inhibit NOX by the follow method: (i) EC will combine with NOX directly; (ii) EC will change the calcium influx, then inhibit the activation of NOX; and (iii) EC can regulate the upstream signaling of NOX, for example, by blocking the combination of ligands with receptors which can promote NOX [10].

### 2.2. Effect of Catechins on GSH and Enzymatic Antioxidants

Synthetized by mucosal cells, GSH is an antioxidant acting as a cellular defense component. It can scavenge free radicals and peroxides [82], and maintain the function of protein sulfhydryl groups facing oxidative stress [30]. The presence of a catechol group in the B ring is essential for catechins to show synergistic effects with GSH in an antioxidant capacity [82].

The overproduction of reactive oxygen nitrogen species (RONS) depletes intracellular GSH. The decrease can stimulate the Nrf2 signaling activation to induce the enhancement of HO-1 and GSH, itself, for the maintenance of homeostasis. However, when the damage from RONS overwhelms the benefit from the activated antioxidant, diseases are induced [58]. By using flow cytometry, Chiou et al. demonstrated that the catechin analogs (ECG, EGCG, AcEGCG) could elevate the amount of GSH in LPS-induced RAW264.7 cells, but not in a dose-dependent manner [58]. However, they found that the low dose of catechins had a highly potent ability compared to the high dose. The researchers proposed that perhaps the low dose of catechins could induce the expression of Nrf2-related GSH-related enzymes, like GPO, resulting in regulating GSH expression. Another experiment proved that EC at 10 mg/kg could induce a higher dose of GSH both in acute and chronic colitis, with relapse in rat models. The researchers inferred that the high levels of GSH were caused by the increase of expression and/or the decrease of depletion induced by EC [30].

On the other hand, in response to the benign oxidative stress, the defense mechanisms will be carried out with an increase in enzymatic antioxidants and other low molecular weight antioxidants [40]. When the body is exposed to the oxidative stress coming from the over-production of ROS for a long time, the defense mechanisms cannot induce enough production of antioxidant enzymes, such as catalase (an H_2_O_2_-specific catalyst), GPO, which can catalyse the reduction of peroxides and act as a barrier against hydroperoxide attack [55], and superoxide dismutases (SOD) [88], which are essential as the sturdy antioxidant defense system by catalysing peroxy anions. It has been demonstrated that this may be the result from the ROS which can inactivate one to several antioxidant enzymes [40]. Additonally, it has been noticed that the deficiency of GPO genes can contribute to the occurrence of IBD in mice [89].

Catechins have been shown to enhance the activity of a number of protective enzymes, resulting in a protective effect [55]. The effect of EGCG intragastrically administered for colitis has been evaluated in the murine model induced by oral administration of DSS. Brückner et al. have revealed that the combination of EGCG and piperine, which was used to raise the bioavailability of EGCG, show a positive antioxidative potential consecutively on the antioxidant enzymes GPO and SOD in immunohistochemical analysis, confirming the notion with only EGCG [40]. The activities of catalase can also be increased by EGCG, EC through decreasing ROS, malonedialdehyde (MDA), and protein carbonyls [61,84], but the exact mechanisms of EGCG on antioxidant enzymes are still unknown [40]. In human HepG2 cells treated with a cocoa polyphenolic extract, which contained EC, ECG, procyanidin B2 and other flavanols, researchers reported that the activities of GPO and glutathione reductase (GR), which can recycle oxidized glutathione back to reduced glutathione, would be enhanced [90].

### 2.3. Effect of Catechins on Cell Infiltration

The main protagonists in IBD include neutrophils, lymphocytes, monocytes, and macrophages. The migration and activation of them into the inflamed tissue depend on the cytokines, chemokines, and adhesion molecules [18]. The cytokines and chemokines are extracellular signaling molecules which mediate cell–cell communication. They can mediate cell proliferation, differentiation, gene expression, migration, immunity, and inflammation. The inflammatory process of IBD includes a large amount of infiltration of T cells, polymorpho- and mononuclear phagocytic leukocytes, which will promote local inflammation and oxidative stress and extend the damage to the mucosa through production of abundant amounts of pro-inflammatory cytokines, chemokines, and reactive oxygen intermediates [3,8,18,40,81], which may further promote cell infiltration to enlarge inflammation in a loop [7]. Mochizuki et al. showed that the migration of neutrophils depended on cytokines secreted by macrophages and mast cells [8]. They also demonstrated that increased levels of newly-recruited monocytes would gather to the inflamed intestinal mucosa from the blood and derive into macrophages and dendritic cells, instead of succumbing to apoptosis after circulation in the blood for several days without specific signals [8,29,63]. In addition, some researchers found that EGCG had an inhibitory effect on endothelial VACM-1, resulting in a decrease of monocyte adhesion to endothelial cells and movement into the inflamed tissue. It was shown that treatment with EGCG in THP-1 decreased gene and protein expression of the monocyte chemotactic protein 1 (MCP-1) and of the MCP-1 receptor (CCR2), inhibiting excessive THP-1 migration to the damaged tissue [9].

The quantities of neutrophil infiltration could be indicated by the activation of myeloperoxidase (MPO), a lysosomal peroxidase enzyme existing most abundantly in neutrophil granulocytes [40]. However, it was previously reported that EGCG could reduce MPO activity, indicating the reduction of neutrophil infiltration [91].

In a study to evaluate the anti-inflammatory and antioxidative effects of EGCG on a DSS-induced murine colitis model by intragastric application, which corresponded to applicable forms for humans, the MPO was tested to assess the leukocyte infiltration. It was observed that the MPO was reduced in piperine plus EGCG in colon tissue. The piperine could not inhibit the neutrophil infiltration alone. However, the EGCG purely failed to eliminate the MPO [40], maybe owing to the low bioavailability. In another experiment regarding the effect of EC on TNBS-induced colitis rat models, EC could not decrease the neutrophil infiltration through any change in the enzyme MPO. It has been implicated by the subjects that the inability may be due to the sustained and extensive damage resulting from so high a concentration of MPO that it is difficult to take effect with any treatment [30].

The study in the TNBS-induced rat colitis by Mochizuki and Hasegawa showed that EGCG could significantly decrease the enzyme MPO and histamine in the distal gut mucosa [8]. It is important that EGCG can suppress the macrophage migration, causing the inhibition of neutrophil migration. The experiment exhibited the increasing mast cells and the high concentration of histamine, both of which could be found in the mucosa of IBD, and were also ameliorated by EGCG in a dose-dependent manner. Thus, the neutrophil infiltration could be inhibited by EGCG, owing to histamine being a kind of chemoattractant of neutrophils [8,9].

### 2.4. Effect of Catechins on Cell Proliferation and Apoptosis

After being stimulated by various factors, such as cytokines, bacterial components, and ROS, CD4^+^ T cells will proliferate and differentiate into kinds of subsets, like Th1 cells, Th2 cells, Th17 cells, and Treg cells, respectively, exhibiting distinct effects to regulate the progress of inflammation in IBD [14,16,92]. The monocytes which can transform to macrophages and dendritic cells play an important role in the inflamed tissue as the main effector cells in the initiation, development, and result of the immune-related response [29,63]. Activated macrophages play important roles in inflammatory diseases via excessive production of inflammatory mediators, such as nitric oxide (NO), and pro-inflammatory cytokines, promoting inflammatory responses [29]. And the anti-inflammatory cytokines, such as IL-4 and IL-10, will promote the monocytes toward apoptosis.

Abundant evidence shows that catechins could benefit the inflamed mucosa of IBD by decreasing the excessive numbers of harmful cells, such as monocytes, and by recovering the physical barrier, such as epithelia, to balance the homeostasis between cell proliferation and cell death [30].

A study conducted by Wu et al. assessed the effect of EGCG on CD4^+^ T cells [93]. With the administration of EGCG and IFN-γ in CD4^+^ T cells (i.e., primary CD4^+^ T cells from C57Bl/6 mice and a human leukemic CD4^+^ T cell line of Hut 78 cells) and non-CD4^+^ T cells (i.e., HepG2 cells), the study indicated that EGCG alternatively enhanced the signal transducer and activator of transcription (STAT) 1 phosphorylation, but inhibited the STAT1 homodimer formation via the Src pathway, but not the Janus kinase (JAK) 1/2 pathway, ultimately promoting the apoptosis of IFN-γ-induced CD4^+^ T cells and benefiting the T cell-related colitis [93]. The research of Kawai et al. revealed that EGCG specifically induced the apoptosis of monocytes [63], administering peripheral blood extraction of mononuclear cells with catechins as the control group. Additionally, EGCG and ECG could significantly promote the apoptosis of mononuclear cells; however, EC and EGC did not. The important agent, which is supposed to be essential in apoptosis, is the common structure of the galloyl group in EGCG and ECG, but not the pyrogallol group, which is present in EGC and EGCG but not in EC and ECG [63]. Its inhibition to mononuclear cells, unlike the glucocorticoid, the function of catechins is not affected by granulocyte-macrophage colony stimulating factor (GM-CSF) and LPS. Kawai et al. also found that EGCG would enhance the component of caspases 3, 8, and 9 content in a dose-dependent manner, which involved two major pathways of apoptosis—the death receptor pathway and the mitochondrial pathway to promote monocytes to die [63].

Epithelial cells belong to the innate physical barriers preventing the attachment of antigens. After being stimulated, epithelial cells can present antigens and secrete an abundance of cytokines to maintain the balance between pro-inflammatory and anti-inflammatory events. Defects in the mucosal barrier contribute to the pathogenesis of IBD [3]. Oligonol, a formulation consisting of 17.6% of catechin-type monomers and 18.6% of proanthocyanidin dimers and trimers, was proved to prevent oxidative stress-induced apoptosis of colonic epithelial cells [7]. In a study to assess the effect of EC on the prevention and treatment of intense inflammation in acute and chronic models of colitis induced by TNBS, the rats were fed with different doses of EC. The researchers found that there was a significant increase of Proliferating Cell Nuclear Antigen (PCNA) and Epidermal Growth Factor (EGF) in the chronic and acute colitis model which was administrated with a dose of 10 mg/kg EC (EC10). The PCNA represent the cells which are in the S phase of the cell cycle. While the EGF can stimulate epithelial cells to proliferate, the study indicated that EC could induce the proliferation of epithelial cells, to recover the intestinal mucosa of colitis, and reduce the relapse lesion. However, when they increased the concentration of the EC, the effect of EC10 would disappear for the pro-inflammatory function of EC [30].

### 2.5. Effect of Catechins on Gap Junctions

Gap junctions, the membrane channels permitting the direct exchange of small water-soluble molecules between adjacently-coupled cells, are important in cellular growth control, differentiation, and apoptosis [42,80,94,95]. When the gap junctional intercellular communication (GJIC) among each cell is down-regulated, the properties to develop into malignant lesions are increased [94,96].

Catechins have shown protective functions to the GJIC which can be down-regulated by various stimuli, such as inflammatory mediators, cytokines, and oxidative stress in various kinds of cells [97]. GTP have the ability to prevent the down-regulation of GJIC in pentachlorophenol-induced mouse hepatocarcinogenesis [97]. EC exhibited positive effects on GJIC in WB-F344 cells treated with 12-*O*-tetradecanoylphorbol-13-acetate (TPA), which has inhibitory effects on GJIC by phosphorylation of the gap junction protein connexin43 (CX43) [98]. ECG could also enhance GJIC in *p*,*p*′-dichlorodiphenyltrichloroethane (DDT)-treated WB-F344 cells in dose-related ways [99]. Hydrogen peroxide-induced inhibition of GJIC was also blocked by treatment with EGCG in liver epithelial cell lines [100], contrary to the view of Lee et al., which brought forward that EGCG could inhibit GJIC and induce phosphorylation of Cx43 [42]. Additionally, EGCG was shown to activate ERK-MAPK, resulting in the down-regulation of CX43 and GJIC in endothelial cells [101]. Furthermore, EGCG could greatly improve the GJIC-inhibitory effects of dimethylnitrosamine in kidney cells [101].

Most investigators believe that catechins have a protective effect on GJIC. Recent studies about the roles of catechins in gap junctions have mainly focused on cardiomyocytes [102], vascular endothelial cells to prevent cardiovascular diseases, lung cells, liver cells [42], and kidney cells [101] etc., about their anti-cancer effect, with less emphasis in intestinal tumors and even IBD. However, studies also showed that catechins at least prevented intestinal damage. It has been proposed EC was shown to up-regulate GJIC between epithelial cells, helping prevent the progression of gastrointestinal lesions from worsening into cancer [30].

### 2.6. Effect of Catechins on Cell Signaling Pathways

#### 2.6.1. Effect of Catechins on NF-κB

**Figure 5:**
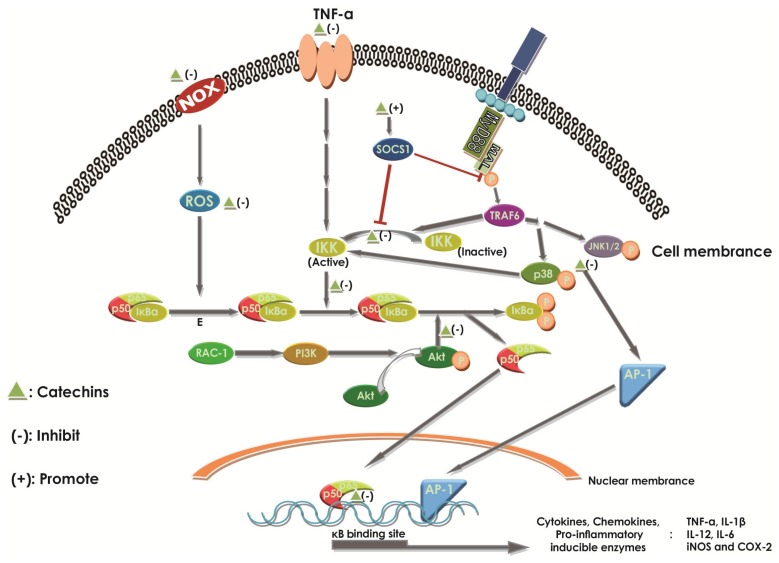
Catechins regulate the activation of NF-κB at multiple levels. Catechins demonstrate direct inhibitory effect on the oxidant or inhibit the NF-κB-related upstream NOX [10]. Also catechins can inhibit NF-κB activation through IKK inactivation by several mechanisms such as up-regulating SOCS1 expression, which could impair inflammation. Catechins can inhibit the activation of NF-κB by regulating the upstream protein kinases JNK1/2, p38, and PI3K/Akt etc. Inside the nucleus, catechins can interact with the DNA-binding site in the NF-κB proteins, thus preventing gene transcription.

Abundant evidence is available to support the notion that the activation and nuclear translocation of NF-κB play an important role in the pathogenisis and development of IBD [3,14,91]. Under the physiological condition, the NF-κB member p65/p50 heterodimer remains an inactive form in the cytoplasm where it forms a complex by bonding to inhibitors, like IκBα [3]. Upon activation by distinct pro-inflammatory stimuli, the NF-κB becomes free through the phosphorylation and degradation of IκBα by IκB kinase (IKK), then translocates to the nucleus and induces transcription of a wide range of genes [3,7,10,18,29,103] in different types of cells, such as macrophages and dendritic cells (Figure 5). Some of these genes encode for several kinds of cytokines, like TNF-α, chemokines, like IL-8, pro-inflammatory enzymes, like cyclooxygenase-2 (COX-2) and inducible nitric oxide synthase (iNOS), cell adhesion molecules, and growth factors [14,104]. The protein product of the genes can be observed at high levels in patients with IBD, too [14]. Generally, NF-κB is used to maintain gut homeostasis. It can help the epithelial cells and immune cells to resist the damage from pathogenic agents, but the excessive activation of NF-κB can amplify the intestinal inflammation then damage the tissue [3]. High levels of NF-κB could be observed in the mucosal cells of IBD patients [18]. In addition, once the NF-κB and related material were inhibited, we found that the progression of IBD was alleviated [104,105].

An increasing number of studies demonstrated that catechins could interact with NF-κB in multiple steps in the activation process (Figure 5). Catechins can disrupt the activation and translocation of NF-κB [14,106]. EGCG can inhibit IκB degradation in a dose-dependent manner by inhibiting AKT phosphorylation, a necessary upstream step, and inhibitor-k kinase activation for NF-κB activation [3,14]. In addition, EGCG can finally induce the degradation of TLR-4 co-factor MyD88 adaptor-like (MAL) protein, which links TLR-4 signaling to NF-κB activation, by enhancing the activation of the suppressor of cytokine signaling 1 (SOCS1), which dampens inflammatory responses as a kind of compensatory anti-inflammatory pathway [14] (Figure 5). Chiou et al. revealed, for the first time, that ECG could significantly inhibit the p-p65 in the nucleus, which represented the activation of NF-κB, in LPS-induced RAW264.7 macrophages by inhibiting the upstream protein kinases c-Jun amino-terminal kinases (JNK) 1/2, p38, and phosphatidylinositol 3-kinase/protein kinase B (PI3K/Akt), or activating the Nrf2 pathway [58]. In the previous study, as Fraga et al. demonstrated, EC could inhibit NF-κB in distinct levels in the activation pathways [10]. EC can act on both the cell membrane and the intracellular targets. For example, EC can suppress the activation of NF-κB by inhibiting NOX and the subsequent superoxide production through reacting directly with the enzyme or inhibiting the combination from ligands to receptors, like TNF-α to their receptors (Figure 5), which triggers NOX activity, and then induces NF-κB. Additionally, EC can directly scavenge free radicals and oxidants, and enter the downstream of NOX to reduce the activation of NF-κB. Even EC can protect NF-κB transcription from interaction of NF-κB with DNA-binding sites in the gene promoters [10] (Figure 5). Experiments showed that in the DSS-induced mouse, the effect of EC and (+)-catechin on NF-κB activation did not perform obviously, because the content of nuclear NF-κB p65 had no significant change after polyphenol-enriched cocoa extract (PCE)-treatment, extracted from cocoa, including EC and (+)-catechin [105]. Therefore, catechins, a subset of polyphenols rich in tea, can significantly suppress the inflammation of IBD by inhibiting the activation of NF-κB through different levels (Figure 5).

#### 2.6.2. Effect of Catechins on MAPKs

**Figure 6:**
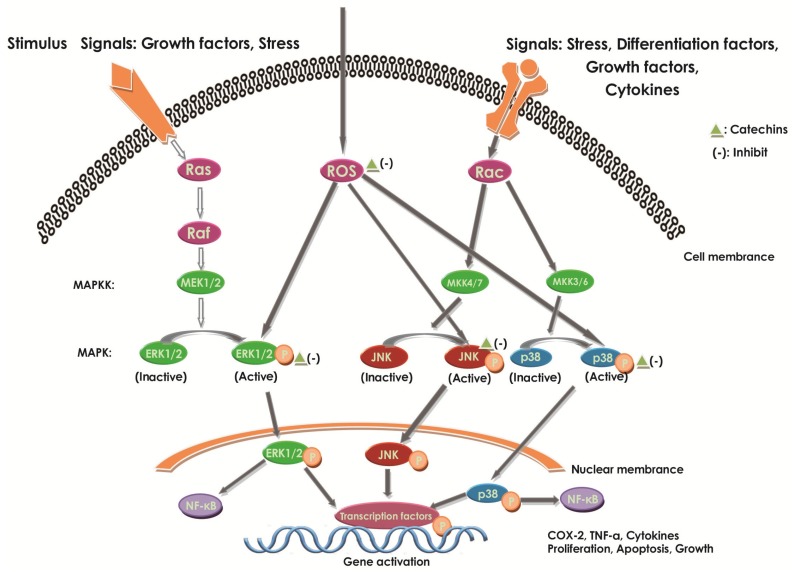
Catechins show an inhibition of the MAPK cascade pathway through the inhibition of the phosphorylation of ERK1/2, JNK, and p38.

Increasing studies proved that MAPK pathways played an important role in the pathogenesis process of IBD. The inhibition of MAPK activation can be a useful treatment to benefit IBD [107]. MAPKs consist of a redox-sensitive family of serine–threonine kinases that mediate fundamental biological processes and cellular responses to external stress signals [29]. MAPKs can be regulated by the MAPK kinases and the MAPK phosphatases through phosphorylation and dephosphorylation, respectively, to active and inactive states. The MAPKs, known as being able to activate NF-κB [28], contains three groups: extracellular signal-regulated kinase (ERK), JNK, and p38 MAP kinases [53] (Figure 6). The inactivation of MAPK phosphatases can lead to persistent activation of JNK and p38, constituting one regulatory point of well-known sensitivity to oxidative stress. 

It was established that MAPKs could regulate the expression of pro-inflammatory mediators [29]. Upon various activation, like LPS, MAPKs could be phosphorylated and then up-regulate the expression of iNOS and COX-2 to promote the inflammation [29]. The p38 MAPK can also be demonstrated to enhance the production of pro-inflammatory cytokines, thus, in turn, stimulating the phosphorylation of p38 MAPK and expanding the inflammation [28]. Additionally, after stimulated by stress, growth factors [108], activated JNK can promote the activation and differentiation of T cells and the expression of pro-inflammatory cytokines [53]. Thus, MAPK’s inhibition on JNK can provide an effective therapy against inflammation, such as IBD [53].

It was proved that EC could reduce the active MAPKs through reduction of oxidant concentration [10]. EGCG can attenuate the ERK of the MAPK pathway in human mast cell lines (HMC-1) [40]. In LPS-induced peritoneal macrophages, it was observed that (+)-catechin would inhibit the phosphorylation of MAPKs [29]. In activated THP-1 human monocyte cell lines, catechins exert different inhibiting abilities on MAPK pathways through regulating p38, JNK, and their phosphorylated forms, reducing oxidative stress and inhibiting a wide range of pro-inflammatory genes [29,109] (Figure 6). Other studies in vivo models of UC showed that (+)-catechin could inhibit the MAPK pathway by depressing JNK and p38 activation and, thus, contribute to the remission of UC [29]. EGCG could inhibit the receptor activator of NF-κB ligand (RANKL)-induced activation of the JNK pathway [110]. EGCG also showed an inhibition of LPS-induced phosphorylation of p38, JNK, and ERK1/2 (Figure 6), in bone marrow-derived macrophages [29]. All could show that the MAPK cascade pathway inhibition by catechins could be critical to the establishment of both anti-inflammatory and protective effects (Figure 6).

The catechin effect on the LPS-induced inflammation in RAW264.7 cells was examined by Liu et al. by using the extract of the lotus leaf [53]. They investigated the anti-inflammatory effect of catechin on iNOS, COX-2, and inflammatory cytokines, such as IL-6 and TNF-α. Furthermore, they revealed that the catechin could block the phosphorylation of JNK, but not ERK and p38. Further, they found that the use of the phospho-JNK inhibitor (SP600125) and catechin could both inhibit the activation of NF-κB. Thus, the results indicated that inflammation could be prevented by catechin, which reacted as the MAPK-inhibitor to inhibit the LPS-induced phospho-JNK, combining with decreasing the NF-κB [53]. EGCG could attenuate the ERK of the MAPK pathway and NF-κB, thus inhibiting the secretion of TNF-α, IL-6, and IL-8 in HMC-1 [40]. A similar result was described by Danesi et al., where the cross-talk between MAPKs and NF-κB played an important role in the inhibition of TNF-α mRNA expression by EGCG in TNF-α over-expression due to IL-23 in kit 225 cells [111]. Moon et al. also reported that EGCG could block both the MAPKs and NF-κB pathways in HT29 cells [112]. Thus, we can conclude that catechins can attenuate the inflammation by inhibiting the cascade of MAPKs and NF-κB. Thus, catechins may act as a therapeutic agent for preventing inflammation [53].

#### 2.6.3. Effect of Catechins on Nrf2

Nrf2 activation seems to play a promising strategy in maintaining active antioxidant pathways in response to oxidative stress-induced inflammation [10,58]. Numerous studies in vivo showed that Nrf2-deficient mice exhibited increased DSS or AOM/DSS-mediated intestinal inflammation and ACF formation and tumors, as compared with Nrf2 wild-type mice [76]. Some cell and animal experiments supported the proposition that Nrf2 could be a novel epigenetic therapeutic intervention used to control various forms of inflammatory disorders, like IBD, and even inflammatory-driven colon cancer [76].

**Figure 7:**
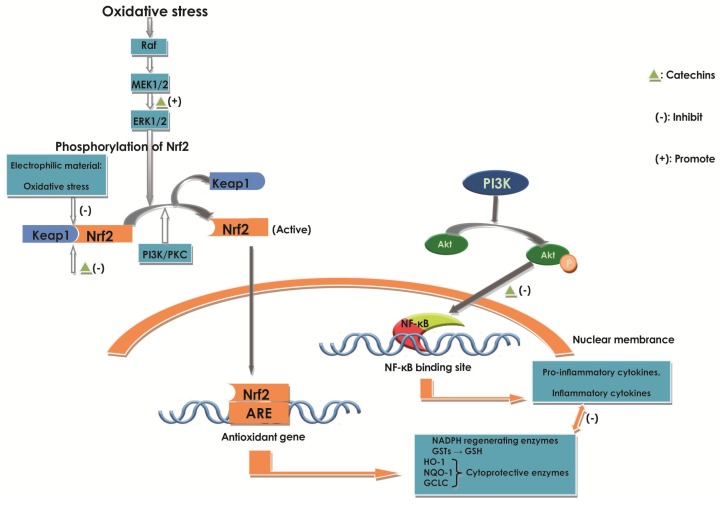
Catechins can activate Nrf2 by enhancing the phosphorylation of ERK1/2, then regulate the activation of Nrf2 by directly inhibiting Keap1-Nrf2 interaction or enhancing ERK1/2 expression. Catechins can regulate the Nrf2 by acting on NF-κB.

Nrf2 is a transcription factor central to the protection of cells against the adverse effects of oxidative and electrophilic stress [10,113]. Usually, Nrf2 remains in an inactive state in the cytosol, binding to the protein kelch-like ECH-associated protein 1 (Keap1), forming a complex [10]. After stimulated by various oxidative stresses, like ROS, this complex would be disrupted to form active Nrf2, leading Nrf2 to translocate to the nucleus and bind to antioxidant response element (ARE) genes, which can encode proteins with cytoprotective functions, like NADPH regenerating enzymes [10,35] (Figure 7).

Various studies proved that activation of Nrf2 could up-regulate NAD (P) H, GSH, and HO-1 expression, which could suppress oxidative stress and inflammatory responses [34,58,76]. Previously, it was revealed that catechins had a significant effect on neurologic damage. For example, EC stimulated the Nrf2 signaling pathway in primary cultures of astrocytes [114].

Other studies with knockdown of Nrf2 mediators and testing various related agents in LPS-activated RAW264.7 murine macrophages showed the similar conclusion. Researchers also proposed that HO-1 induction was regulated by the complex cross-talk of MAPKs, NF-κB, and Nrf2. The results provided new evidence that HO-1 induction was involved in ERK1/2 signal-mediated cross-talk between NF-κB and Nrf2/ARE transcriptional activity, suggesting that NF-κB-regulated pro-inflammatory signaling was related to upstream p38, JNK, and PI3k/Akt pathways, which occurred earlier than Nrf2/ARE-regulated antioxidant signaling under LPS-stimulated oxidative stress by enhancing NF-κB and disrupting the keap1-Nrf2 [58]. It was also proved that ECG might inhibit the NF-κB by suppressing the LPS-induced activation of the p38, JNK, and PI3k/Akt pathways. At the same time, ECG can enhance the phosphorylation of ERK1/2, then regulate the activation of Nrf2, resulting in the significant increase of GSH and HO-1 expression [58], protecting cells from oxidative stress and other toxic agents [7] (Figure 7). Additionally, acting as the direct Keap1-Nrf2 interaction inhibitor, ECG is a better option to activate Nrf2 for inflammatory disease than ROS, which can contribute serious damage to tissue by not only enhancing the HO-1 expression [58] (Figure 7). Furthermore, the decrease of NF-κB is a benefit to Nrf2 expression. The study also pointed out that the ERK1/2 of the MAPK pathway might play a dual role in the mediation of NF-κB and Nrf2 signaling with pro-inflammatory and antioxidant gene regulation [58]. Therefore, catechins can contribute to inhibiting the inflammation and oxidative stress by acting as a potential Nrf2 activator to improve antioxidant agents, like HO-1 expression and NF-κB repressors (Figure 7). The study supported the idea that enhancing Nrf2 expression could contribute to a potential treatment to prevent inflammation and oxidative damage in the intestinal epithelium during IBD [76].

Increasing evidence shows that there is a cross-talk between NF-κB, MAPKs, and Nrf2 pathways to antioxidants [58,76]. It was reported that NF-κB could directly repress Nrf2 signaling at the transcription level by enhancing the concentration of co-repressors or competing against Nrf2 for coactivators like CREB binding protein (CBP) [115,116], which was essential for transcription and localization of Nrf2 [76]. Nrf2 could attenuate NF-κB transcriptional activity in inflammatory responses [58]. Catechins may inhibit the oxidative stress damage and inflammation through the cross-talk between them. A study conducted by Chiou et al. evaluated the chemopreventive and molecular mechanism of dietary administration with AcEGCG, which acted as a prodrug of EGCG to improve the biological activity and bioavailability of EGCG, and EGCG in DSS-induced colitis in mice [76]. The results indicated that AcEGCG administration was more effective than EGCG in suppressing DSS-induced symptoms of colitis and colon cancer associated colon colitis [76]. The results indicated that AcEGCG reduced p65 phosphorylation and NF-κB-DNA-binding activity, but did not affect CBP activity. The protein level of Nrf2 was significantly escalated in the AcEGCG-treated mucosa dose-dependently than the control, depending on histone acetyltransferase (CBP/p300) activity, owing to the NF-κB inactivation and ERK (p44/42) MAPK phosphorylation. The increase of Nrf2 resulted in enhancing antioxidant enzyme (HO-1) expression, which contributed to antioxidant, anti-apoptotic, and anti-inflammatory properties [58].

#### 2.6.4. Effect of Catechins on STAT1 and STAT3

It was noted that transcription factors—STAT1 and STAT3 were implicated in the pathogenesis of IBD [105]. In the neutrophils and monocytes of the intestinal lamina propria of IBD patients, it was observed that there was an increased expression and activation of STAT1, which was greater in UC than in CD [105,117]. The STAT3 in IBD was proved to be important according to animal and human IBD studies. It takes on a different role in the immune system, connecting with the pathogenesis of IBD [118]. After stimulation, the active STAT1 can form homodimers, which are translated into the nucleus and bind to gamma activated sequence (GAS) to regulate the expression of genes [14,93]. After stimulation by cytokines, such as IL-6, TNF-α, and growth factors, the tyrosine residue 705 in the C-terminal transactivation domain (TAD) can be phosphorylated, forming the activated dimerization of STAT3, and then the active ones will translate into the nucleus and up-regulate various genes, such as the pro-inflammatory enzymes and mediators [7,14,118].

EGCG can inhibit JAK1/2, resulting in suppressing the activation of STAT1 and STAT3 from forming the STAT1–STAT1, STAT1–STAT3, and STAT3–STAT3 homodimers, respectively induced by IFN-γ, IL-27, and IL-6 [14]. It was proved that EGCG inhibited STAT1 activation in many cell lines like HePG2 cells. For example, EGCG could suppress the JAK/STAT1 pathway, then suppress indoleamine 2,3-dioxygenase expression in IFN-γ-induced murine dendritic cells [93]. However, on the contrary, EGCG could increase the activation of STAT1 in IFN-γ-induced CD4^+^ T cells, such as splenic CD4^+^ T cells and Hut 78 cells, but it still suppressed the STAT1 homodimer formation and inhibited the downstream signaling: the mRNA expression of the IFN-γ-induced pro-inflammatory gene, chemokine (C–X–C motif) ligand 9 (CXCL9) [93]. EGCG could still inhibit JAK1/2, resulting in the decrease of STAT3, then inhibit the pro-inflammatory signaling in CD4^+^ T cells [93].

Increasing studies support that the regulation in levels of over-activation of NF-κB and STAT3 by dietary or medicinal plants, like lychee, is effective in inhibiting IBD [7,119]. Just like oligonol, it mainly consists of catechin-type oligomers derived from lychee fruit extract, and could inhibit STAT3, NF-κB, and the cross-talk between them, benefiting DSS-induced colitis in mice [7]. Curcumin could also inhibit inflammation by blocking NF-κB and STAT3 pathways, too [59]. In addition, pure gallic acid exerts anti-inflammatory effects through the suppression of both p65-NF-κB and IL-6/p-STAT3 tyrosine 705 activation in DSS-induced UC in murine models, reducing the colonic MPO activity and expression of inflammatory mediators, like iNOS, COX-2, and pro-inflammatory cytokines, and the reduction of the disruption of the colonic architecture [119]. However, in a study of DSS-induced acute UC in mice, oral administration of PCE, which contains EC, procyanidin B2, catechin, and procyanidin B1, could decrease both STAT1 and STAT3 phosphorylation levels with a slight effect on NF-κB, resulting in reduced cytokine production of IL-6 and COX-2, reduced tissue damage and neutrophil infiltration in vivo [105].

### 2.7. Effect of Catechins on Intestinal Flora

The human gut bacteria communities can be divided into different enterocytes (*Bacteroides*, *Prevotella*, or *Ruminococcus*) by metagenomics analysis [6]. The majority of human gut microbiota contain: the *Bacteroidetes* phylum, the *Firmicutes* phylum [120], the *Actinobacteria* phylum, the *Proteobacteria* phylum, and the *Verrucomicrobia* phylum [121]. While the two former phyla contribute to 90% of the classes of the normal microbiome [6], the composition of microbiota in adults is stable and has a large inter-individual variation [121], which can be influenced by diet, BMI, age, intestinal diseases and medication, particularly antibiotics [65,121,122].

Accompanied by the enrichment of molecular techniques, such as the advent of culture-independent, enormous studies, including the NIH sponsored Human Microbiome Project for the human microbiome change in health or disease, indicated that the imbalance of intestinal microflora, its metabolites, and host susceptibility genes, as well as the host intestinal mucosa innate or acquired immune response played an important role in the aetiopathogenesis of IBD [5,6].

The gut microbiota is altered in the IBD patient as a whole. In recent years, researchers suggested that the breakdown of the gut microbiome and the coordination of host-microbial mutualism became the key progress in the development of IBD [6]. Among the diversification of microbiota, the decrease in the amount and diversity of the *Firmicutes* phylum, like obligate anaerobes, is the most obvious, especially *Faecalibacterium prausnitzii*. For the *Bacteroidetes* phylum, there are different views about their increase or decrease [123,124]. While the increasing *Proteobacteria* phylum, including facultative anaerobes, is shown to induce the initiation of chronic inflammatory IBD [6]. Even *Escherichia coli*, consisting of a pathogenic subset found in ileal CD, makes sense in the pathogenesis of CD [6]. However, *bacteroides* can interact with Treg cells and macrophages, stimulating the production of anti-inflammatory cytokine IL-10 [5]. These indicate that the changes in composition of the gut microbiome constituting the host-microbiome ecology are the core influence in IBD, and the adjustment of intervention to the microbiome component may be an approach to IBD.

Several human trials revealed that by suppression of the growth of pathogenic gut bacteria and regulation of inflammation of the bowel, flavonoid (which contains of catechins) intake benefits gut health [125]. Increasing studies indicated that the catechins belonging to polyphenols possessed antimicrobial ability [63,120], in accordance with the conclusion proposed by Archana et al. [126]. Catechins could significantly kill certain pathogenic bacteria, like *Clostridium perfringens*, *Erwinia*, *Pseudomonas*, *Clavibacter*, *Xanthomonas*, *Agrobacterium* spp. [38], *Staphylococcus* spp., *Vibrio parahaemolyticus*, *Bacillus cereus*, and *Plesiomonas shigelloides*, but have less effect in promoting the beneficial bacteria, like *Bifidobacterium* spp. in human studies [36,120]. Xue et al. revealed that catechins could significantly repress the growth of *Bacteroidetes* and *Firmicutes* and down-regulated the rate of *Bacteroidetes* to *Firmicutes* in vitro [120]. Different to the phenomenon observed by Xue et al., Rastmanesh et al. argued that polyphenols had a biased promoting effect on *Bacteroidetes*, but a biased inhibiting property on *Firmicutes* [127]. To some extent, catechins were capable of enhancing several genera of bacteria, including *Bifidobacterium* spp., *Enterococcus* spp., *Streptococcus* spp., and *Collinsella* spp. [120]. Investigations by Amarowicz et al. indicated that the antibacterial functions of catechins are dose-dependent [38]. The EC and EGC had no antimicrobial property against *E. coli* K12 at low doses, being consistent with (+)-catechin by 1000 µM [128]. Even the antibacterial property of EGCG against phytopathogenic bacteria was stronger than EC by 10–20 times, but it exerted slight power at low doses [38].

**Figure 8:**
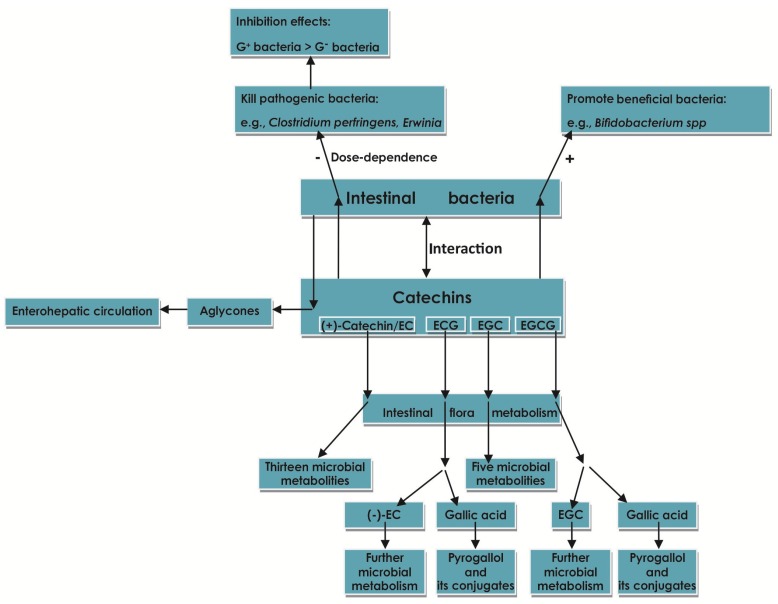
The interaction between catechins and intestinal microflora.

The toxicity of catechin, which has many diastereoisomers like EC and ECG, to bacteria membranes, extracellular proteins, DNA, and morphology was analyzed by Fathima et al., using Gram-positive bacteria (*B. subtilis*) and Gram-negative bacteria (*E. coli*). It was demonstrated that catechin could benefit bacteria in a dose of about 10 μM in *B. subtilis* and about 12 μM in *E. coli*. If the concentration was higher than that, catechin possessed a negative function on bacteria through the oxidative damage resulting in the change in membrane permeabilization and membrane liposomes [36]. This conclusion shows that catechin has a dual function that both benefits and harms to bacteria in a dose-dependent manner, which is consistent with a previous study. The authors also revealed that the inhibition of catechin against bacteria was more obvious in the Gram-positive bacteria (*B. subtilis*) than Gram-negative bacteria (*E. coli*) in all of the permeabilization changes, DNA damage, and stress protein expression [36]. Catechins have a stronger affinity to the peptidoglycan of Gram-positive bacteria than the negatively-charged liopolysaccharides in the membrane of Gram-negative bacteria, resulting in greater permeation, absorption of catechins, and more injury to the cell membrane in Gram-positive bacteria [129,130]. Additionally, catechins can cut the supercoiled DNA into open linear or circular DNA, acting like restriction enzymes in *B. subtilis* DNA, but not in *E. coli* DNA. The extracellular proteins can also be elevated in *B. subtilis* than *E. coli* when both are treated with catechin [36]. In addition, polyphenols can influence gut microbiota, and the gut microbiota can also have an influence on the polyphenols (Figure 8), such as cleaving it to be a kind of energy foundation, especially for *bacteroidetes*. This may be one of the reasons for the change in intestinal microbiota [35].

Indeed, there is significant evidence to prove that polyphenols could not only influence gut microbiota to maintain body health, but also that gut microbial metabolites of polyphenols, like catechins, had an important influence on human health, which might benefit IBD, too [35,119] (Figure 8). It was demonstrated that the existence of the enterohepatic recycling scenario, and the glycosidase and glucuronidase activity of gut microbes, made it possible for gut bacteria to enhance the bioavailability of polyphenols [35,70,131]. Microbial glycosidases excreted by *Enterococcus avium* (LY1) are able to catalyze the hydrolysis of the flavonoid glycosides into aglycones, which are then rapidly absorbed and further metabolized in the liver, which are then excreted back into the lumen [70], which might apply to catechins, too. A general role applicable to the general microbial metabolism of GTCs (green tea catechins) is the following: 1. Under the cleavage of an ether bond –O–, The C-ring is going through ring-fission. Additionally, gallated catechins are broken down; 2. On the B-ring, lactones conform to partial dehydroxylation; 3. They can conform to phenolic acids and some of their conjugate derivatives, and benzoic acids and some of their conjugates [67]. The main gut microbial metabolites of catechins appear to be 3-hydroxyphenylacetic acid, 3-hydroxyphenylpropionic acid, 3,4-dihydroxyphenylaceticacid, and 3-hydroxyphenyl-γ-valerolactone [35,132]. In vitro and in vivo animal and human systems, thirteen microbial metabolites of (+)-catechin and (–)-EC have been revealed: 1-(3′,4′-dihydroxyphenyl)-3-(2′′,4′′,6′′-trihydroxyphenyl)-propan-2-ol, 1-(3′-hydroxyphenyl)-3-(2′′,4′′,6′′-trihydroxyphenyl)-propan-2-ol, 5-(3′,4′-dihydroxyphenyl)-γ-valeric acid, δ-(3′,4′-dihydroxyphenyl)-γ-valerolactone, δ-(3′-hydroxyphenyl)-γ-valerolactone, 5-(3′,-hydroxyphenyl)-γ-valeric acid, δ-(4′-hydroxy-3′-methoxyphenyl)-γ-valerolactone, 3′,4′-dihydroxybenzoic acid, m-hydroxyphenylpropionic acid, 4′-hydroxy-3′-methoxybenzoic acid (vanillic acid), m-hydroxyphenylhydracrylic acid, m-hydroxybenzoic acid, and m-hydroxyhippuric acid [65,67]. After hydrolysis by intestinal flora, ECG will form gallic acid and (–)-EC. Both gallic acid and (–)-EC will undergo further microbial metabolism. Actually, gallic acid will become pyrogallol and pyrogallol conjugates. As for EGCG, it will form EGC and gallic acid, then undergo further microbial metabolism; Usually, EGC can form 1-(3′,4′,5′-trihydroxyphenyl)-3-(2′′,4′′,6′′-trihydroxyphenyl)-propan-2-ol, δ-(3′,4,5′′-trihydroxyphenyl)-r-valerolactone, δ-(3′,4′-dihydroxyphenyl)-r-valerolactone, δ-(3′,5′-dihydroxyphenyl)-r-valerolactone, and δ-(3′,5′-dihydroxyphenyl)-r-valerolactone-3′-glucuronide [67]. At the same time, an abundance of papers revealed the exact enzymes and microbe species breaking down from catechins to metabolites, trying to make sure “what they do” and “who are there”, what bacteria are doing with respect to catechins [35,133]. For example, *Gordonibacter faecihominis* sp. Nov., a novel actinobacterial strain belonging to the genus *Gordonibacter*, isolated from human feces, is capable of dehydroxylating (+)-catechin derivatives [133]. Another human intestinal bacterium *Eggerthella* sp. CAT-1 can cleave the C-ring and dehydroxylate the B-ring of both (+)-catechin and (–)-EC [134]. Wang et al. proved that a human intestinal bacterium *Eubacterium* sp. Strain SDG-2 would cleave the C-ring of (3*R*)-isomers, like (–)-catechin and (–)-EC, GC and EGC, and (3*S*)-isomers, like (+)-catechin (2*R*,3*S*) and (+)-EC (2*S*,3*S*), to give 1′,3′-diphenylpropan-2-ol derivatives, and had the ability of p-dehydroxylation in the B-ring of (3*R*) isomers, but not of (3*S*) isomers [135]. Bacterial enzymes, including the enterohepatic circulation-related ones, can induce many enzymatic reactions including hydrolysis, hydrogenation, dehydroxylation, oxidation, ring cleavage, decarboxylation, and rapid deconjugation, resulting in the microbial metabolites of catechins [67]. For example, through the reaction with a complex with peroxyl iron of dioxygenase, catechin can finally convert into 3, 4-dihydroxyphenyl acetic acid [136], and β-glucosidase, isolated from *Novosphingobium* sp., revealed transglycosylation activity toward (+)-catechin, synthesizing catechin glycosides [137]. However, the species of microorganisms that take effect, the corresponding enzymes, and the specific mechanisms, even the exact relationships require further exploration.

### 2.8. Effect of Catechins on Tight Junctions (TJ)

TJ, a dynamic multifunctional complex that forms a seal between adjacent epithelial cells, in which zonula occludens- (ZO-) 1 protein plays a central role in TJ functions connecting the occludin and claudin to other proteins, playing an important role in the intestinal barrier [5,138,139]. The change of TJ regulators and components, and the related barrier permeability, will exacerbate tissue damage in IBD [140]. Several cell studies showed that catechins might benefit IBD by improving TJ stability in epithelium cells [5,139,141].

Contreras et al. proved that EC could inhibit TNF-α-induced permeabilization of Caco-2 cell monolayers through inhibiting the decrease and redistribution of ZO-1 protein, preventing IBD from becoming worse [139]. Additionally, Carrasco-Pozo et al. proved that several polyphenols, including EGCG, had a similar function, inhibiting the redistribution of the ZO-1 protein and the decreased expression of ZO-1 and occludin induced by indomethacin in a Caco-2-based model [5,141].

## 3. Conclusions

In summary, we propose that catechins have the slight potential for use in ameliorating IBD and related abnormal conditions, with its known anti-inflammatory, antioxidative, and anti-bacteria activation. Importantly, as antioxidants, catechins can suppress the damage to cells and tissues originating from the imbalance between the oxidation system and antioxidant system by the enhancement of the antioxidant, the related enzymes, and the direct or indirect antioxidative effects, which depend on the concentration. Catechins can also inhibit the infiltration and proliferation of immune-related cells and regulate inflammation and oxidative reactions by interaction with a plurality of inflammation-related oxidative stress-related pathways, such as NF-κB, MAPKs, Nrf2, and STAT1/3 pathways, contributing to the decrease of the production, secretion, and reaction of cytokines, chemokines, pro-inflammatory cytokines, adhesion molecules, and inflammatory-related enzymes, like iNOS and COX-2. Catechins may prevent gastrointestinal lesions from worsening into cancer through the regulation of cell gap junctions and the improvement of the expression of TJ in the epithelium. The regulation of inflammation may help benefit inflammation in IBD. Finally, catechins maybe also stabilize intestinal flora, helping the recovery of IBD.

However, there is a very limited number of studies, either in the animal models of IBD or in IBD patients, which may help prove the biological activities. More studies are needed to further explore the exact activities and possible mechanisms, even down to the molecular level. Additionally, more laboratory and clinical trials are needed to investigate the appropriate clinical application, the optimal doses in the case of low bioavailability, but high doses exposed in the gut, and the balance of anti-inflammatory and pro-inflammatory properties.

## References

[1] Podolsky D.K. (2002). Inflammatory bowel disease. N. Engl. J. Med..

[2] da Silva M.S., Sánchez-Fidalgo S., Talero E., Cárdeno A., da Silva M.A., Villegas W., Souza Brito A.R., de La Lastra C.A. (2010). Anti-inflammatory intestinal activity of *Abarema cochliacarpos* (Gomes) Barneby & Grimes in TNBS colitis model. J. Ethnopharmacol..

[3] Biasi F., Astegiano M., Maina M., Leonarduzzi G., Poli G. (2011). Polyphenol Supplementation as a Complementary Medicinal Approach to Treating Inflammatory Bowel Disease. Curr. Med. Chem..

[4] Larsen S., Bendtzen K., Nielsen O.H. (2010). Extraintestinal manifestations of inflammatory bowel disease: Epidemiology, diagnosis, and management. Ann. Med..

[5] Kaulmann A., Bohn T. (2016). Bioactivity of Polyphenols: Preventive and Adjuvant Strategies toward Reducing Inflammatory Bowel Diseases—Promises, Perspectives, and Pitfalls. Oxidative Med. Cell. Longev..

[6] Hold G.L., Smith M., Grange C., Watt E.R., EI-Omar E.M., Mukhopadhya I. (2014). Role of the gut microbiota in inflammatory bowel disease pathogenesis: What have we learnt in the past 10 years?. World J. Gastroenterol..

[7] Yum H.W., Zhong X., Jin P., Na H.K., Kim N., Lee H.S., Surh Y.J. (2013). Oligonol Inhibits Dextran Sulfate Sodium-Induced Colitis and Colonic Adenoma Formation in Mice. Antioxid. Redox Signal..

[8] Mochizuki M., Hasegawa N. (2010). (–)-Epigallocatechin-3-Gallate Reduces Experimental Colon Injury in Rats by Regulating Macrophage and Mast Cell. Phytother. Res..

[9] Melgarejo E., Medina M.Á., Sánchez-Jiménez F., Urdiales J.L. (2010). Targeting of histamine producing cells by EGCG: A green dart against inflammation?. J. Physiol. Biochem..

[10] Fraga C.G., Oteiza P.I. (2011). Dietary flavonoids: Role of (−)-epicatechin and related procyanidins in cell signaling. Free Radic. Biol. Med..

[11] Buell M.G., Berin M.C. (1994). Neutrophil-Independence of the Initiation of Colonic Injury. Comparison of Results from Three Model of Experimental Colitis in the Rat. Dig. Dis. Sci..

[12] Yamada T., Zimmerman B.J., Specian R.D., Grisham M.B. (1991). Role of neutrophils in acetic acid-induced colitis in rats. Inflammation.

[13] Pull S.L., Doherty J.M., Mills J.C., Gordon J.I., Stappenbeck T.S. (2005). Activated macrophages are an adaptive element of the colonic epithelial progenitor niche necessary for regenerative responses to injury. Proc. Natl. Acad. Sci. USA.

[14] Dryden G.W., Lam A., Beatty K., Qazzaz H.H., Mcclain C.J. (2013). A Pilot Study to Evaluate the Safety and Efficacy of an Oral Dose of (–)-Epigallocatechin-3-Gallate-Rich Polyphenon E in Patients with Mild to Moderate Ulcerative Colitis. Inflamm. Bowel Dis..

[15] Wang S., Zhang Y., Saas P., Wang H., Xu Y., Chen K., Zhong J., Yuan Y., Wang Y., Sun Y. (2015). Oridonin exerts therapeutic effect by suppressing Th1/Th17 simultaneously in a mouse model of Crohn’s disease. J. Gastroenterol. Hepatol..

[16] Hirota K., Martin B., Veldhoen M. (2010). Development, regulation and functional capacities of Th17 cells. Semin. Immunopathol..

[17] Seiderer J., Elben I., Diegelmann J., Glas J., Stallhofer J., Tillack C., Pfenning S., Jürgens M., Schmechel S., Konrad A. (2008). Role of the Novel Th17 Cytokine IL-17F in Inflammatory Bowel Disease (IBD): Upregulated Colonic IL-17F Expression in Active Crohn’s Disease and Analysis of the IL-17F, p.His161arg Polymorphism in IBD. Inflamm. Bowel Dis..

[18] Rosillo M.A., Sanchez-Hidalgo M., Cárdeno A., Lastra C.A.D.L. (2011). Protective effect of ellagic acid, a natural polyphenolic compound, in a murine model of Crohn’s disease. Biochem. Pharmacol..

[19] Tsuji N.M. (2006). Antigen-Specific CD4^+^ Regulatory T cells in the Intestine. Inflamm. Allergy Drug Targets.

[20] Gupta S.C., Tyagi A.K., Deshmukh-Taskar P., Hinojosa M., Prasad S., Aggarwal B.B. (2014). Downregulation of tumor necrosis factor and other proinflammatory biomarkers by polyphenols. Arch. Biochem. Biophys..

[21] Ben-horin S., Chowers Y. (2014). Tailoring anti-TNF therapy in IBD: Drug levels and disease activity. Nat. Rev. Gastroenterol. Hepatol..

[22] Yanai H., Lichtenstein L., Assa A., Mazor Y., Weiss B., Levine A., Ron Y., Kopylov U., Bujanover Y., Rosenbach Y. (2015). Levels of Drug and Antidrug Antibodies Are Associated With Outcome of Interventions After Loss of Response to Infliximab or Adalimumab. Clin. Gastroenterol. Hepatol..

[23] Howell H.R. (2008). Ulcerative colitis: Achieving and maintaining remission. US Pharm..

[24] Faubion W.A., Loftus E.V., Harmsen W.S., Zinsmeister A.R., Sandborn W.J. (2001). The Natural History of Corticosteroid Therapy for Inflammatory Bowel Disease: A Population-Based Study. Gastroenterology.

[25] Farzaei M.H., Rahimi R., Abdollahi M. (2015). The Role of Dietary Polyphenols in the Management of Inflammatory Bowel Disease. Curr. Pharm. Biotechnol..

[26] Koláček M., Muchová J., Dvořáková M., Paduchová Z., Žitňanová I., Čierna I., Országhová Z., Székyová D., Jajcaiová-Zedníčková N., Kovács L. (2013). Effect of natural polyphenols (Pycnogenol) on oxidative stress markers in children suffering from Crohn’s disease - a pilot study. Free Radic. Res..

[27] Hanai H., Iida T., Takeuchi K., Watanabe F., Maruyama Y., Andoh A., Tsujikawa T., Fujiyama Y., Mitsuyama K., Sata M. (2006). Curcumin Maintenance Therapy for Ulcerative Colitis: Randomized, Multicenter, Double-Blind, Placebo-Controlled Trial. Clin. Gastroenterol. Hepatol..

[28] Kook S.H., Choi K.C., Cho S.W., Cho H.K., Lee K.D., Lee J.C. (2015). Catechin-7-*O*-β-d-glucopyranoside isolated from the seed of *Phaseolus calcaratus* Roxburgh ameliorates experimental colitis in rats. Int. Immunopharmacol..

[29] Sánchez-Fidalgo S., Da Silva S.M., Cárdeno A., Aparicio-Soto M., Salvador M.J., Frankland Sawaya A.C., Souza-Brito A.R.M., Alarcón de la Lastra C. (2013). *Abarema cochliacarpos* reduces LPS-induced inflammatory response in murine peritoneal macrophages regulating ROS-MAPK signal pathway. J. Ethnopharmacol..

[30] Vasconcelos P.C., Seito L.N., Di S.L., Akiko H.C., Ch P. (2012). Epicatechin Used in the Treatment of Intestinal Inflammatory Disease: An Analysis by Experimental Models. Evid. Based Complement. Altern. Med..

[31] Xie G., Ye M., Wang Y., Ni Y., Su M., Huang H., Qiu M., Zhao A., Zheng X., Chen T. (2009). Characterization of Pu-erh Tea Using Chemical and Metabolic Profiling Approaches. J. Agric. Food Chem..

[32] Wang D., Xiao R., Hu X., Xu K., Hou Y., Zhong Y., Meng J., Fan B., Liu L. (2010). Comparative Safety Evaluation of Chinese Pu-erh Green Tea Extract and Pu-erh Black Tea Extract in Wistar Rats. J. Agric. Food Chem..

[33] Danila A.M., Kotani A., Hakamata H., Kusu F. (2007). Determination of Rutin, Catechin, Epicatechin, and Epicatechin Gallate in Buckwheat *Fagopyrum Esculentum* Moench by Micro-High-Performance Liquid Chromatography with Electrochemical Detection. J. Agric. Food Chem..

[34] Satoko A., Atsushi N., Mari M., Mariko U., Akira M. (2012). Effects of anthocyanin-rich tea “Sunrouge” on dextran sodium sulfate-induced colitis in mice. BioFactors.

[35] Stevens J.F., Maier C.S. (2016). The chemistry of gut microbial metabolism of polyphenols. Phytochem. Rev..

[36] Fathima A., Rao J.R. (2016). Selective toxicity of Catechin—A natural flavonoid towards bacteria. Appl. Microbiol. Biotechnol..

[37] Anderson R.F., Fisher L.J., Hara Y., Harris T., Mak W.B., Melton L.D., Packer J.E. (2001). Green tea catechins partially protect DNA from ^·^OH radical-induced strand breaks and base damage through fast chemical repair of DNA radicals. Carcinogenesis.

[38] Amarowicz R., Pegg R.B., Bautista D.A. (2000). Antibacterial activity of green tea polyphenols against *Escherichia coli* K12. Mol. Nutr. Food Res..

[39] Muzolf-Panek M., Gliszczyńska-Swigło A., de H.L., Aarts J.M., Szymusiak H., Vervoort J.M., Tyrakowska B., Rietjens I.M. (2008). Role of Catechin Quinones in the Induction of EpRE-Mediated Gene Expression. Chem. Res. Toxicol..

[40] Brückner M., Westphal S., Domschke W., Kucharzik T., Lügering A. (2012). Green tea polyphenol epigallocatechin-3-gallate shows therapeutic antioxidative effects in a murine model of colitis. J. Crohns. Colitis.

[41] Pan M.H., Chiou Y.S., Wang Y.J., Ho C.T., Lin J.K. (2011). Multistage carcinogenesis process as molecular targets in cancer chemoprevention by epicatechin-3-gallate. Food Funct..

[42] Lee K.W., Lee H.J. (2016). The roles of polyphenols in cancer chemoprevention. BioFactors.

[43] Silva M.S.D., Almeida A.C.A.D., Faria F.M.D., Luiz-Ferreira A., Silva M.A.D., Vilegas W., Pellizzon C.H., Brito C.H.P. (2010). Abarema cochliacarpos: Gastroprotective and Ulcer-Healing Activities. J. Ethnopharmacol..

[44] Fraga C.G., Galleano M., Verstraeten S.V., Oteiza P.I. (2010). Basic biochemical mechanisms behind the health benefits of polyphenols. Mol. Asp. Med..

[45] Scalia S., Marchetti N., Bianchi A. (2013). Comparative Evaluation of Different Co-Antioxidants on the Photochemical- and Functional-Stability of Epigallocatechin-3-gallate in Topical Creams Exposed to Simulated Sunlight. Molecules.

[46] Lambert J.D., Hong J., Yang G.Y., Liao J., Yang C.S. (2005). Inhibition of carcinogenesis by polyphenols: Evidence from laboratory investigations. Am. J. Clin. Nutr..

[47] Yang C.S., Maliakal P., Meng X. (2002). Inhibition of Carcinogenesis by Tea. Annu. Rev. Pharmacol. Toxicol..

[48] Yang C.S., Wang X., Lu G., Picinich S.C. (2009). Cancer prevention by tea: Animal studies, molecular mechanisms and human relevance. Nat. Rev. Cancer.

[49] Xiao H., Hao X., Simi B., Ju J., Jiang H., Reddy B.S., Yang C.S. (2008). Green tea polyphenols inhibit colorectal aberrant crypt foci (ACF) formation and prevent oncogenic changes in dysplastic ACF in azoxymethane-treated F344 rats. Carcinogenesis.

[50] Guan F., Liu A.B., Li G., Yang Z., Sun Y., Yang C.S., Ju J. (2012). Deleterious Effects of High Concentrations of (–)-Epigallocatechin-3-Gallate and Atorvastatin in Mice With Colon Inflammation. Nutr. Cancer.

[51] Jung Y.D., Ellis L.M. (2001). Inhibition of tumour invasion and angiogenesis by epigallocatechin gallate (EGCG), a major component of green tea. Int. J. Exp. Pathol..

[52] Jankun J., Selman S.H., Swiercz R., Skrzypczak-Jankun E. (1997). Why drinking green tea could prevent cancer. Nature.

[53] Liu S.H., Lu T.H., Su C.C., Lay I.S., Lin H.Y., Fang K.M., Ho T.J., Chen K.L., Su Y.C., Chiang W.C. (2014). Lotus Leaf (*Nelumbo nucifera*) and its Active Constituents Prevent Inflammatory Responses in Macrophages via JNK/NF-κB Signaling Pathway. Am. J. Chin. Med..

[54] Shimizu M., Shirakami Y., Sakai H., Adachi S., Hata K., Hirose Y., Tsurumi H., Tanaka T., Moriwaki H. (2008). (–)-Epigallocatechin Gallate Suppresses Azoxymethane-Induced Colonic Premalignant Lesions in Male C57BL/KsJ-*db/db* Mice. Cancer Prev. Res..

[55] Rodríguezramiro I., Martín M.Á., Ramos S., Bravo L., Goya L. (2011). Comparative effects of dietary flavanols on antioxidant defences and their response to oxidant-induced stress on Caco2 cells. Eur. J. Nutr..

[56] Hara Y. (1997). Influence of Tea Catechins on the Digestive Tract. J. Cell. Biochem. Suppl..

[57] Kim M., Murakami A., Miyamoto S., Tanaka T., Ohigashi H. (2010). The modifying effects of green tea polyphenols on acute colitis and inflammation-associated colon carcinogenesis in male ICR mice. BioFactors.

[58] Chiou Y.S., Huang Q., Ho C.T., Wang Y.J., Pan M.H. (2016). Directly interact with Keap1 and LPS is involved in the anti-inflammatory mechanisms of (–)-epicatechin-3-gallate in LPS-induced macrophages and endotoxemia. Free Radic. Biol. Med..

[59] Kunnumakkara A.B., Bordoloi D., Padmavathi G., Monisha J., Roy N.K., Prasad S., Aggarwal B.B. (2016). Curcumin, the Golden Nutraceutical: Multitargeting for Multiple Chronic Diseases. Br. J. Pharmacol..

[60] Kang W.S., Lim I.H., Yuk D.Y., Chung K.H., Park J.B., Yoo H.S., Yun Y.P. (1999). Antithrombotic Activities of Green Tea Catechins and (–)-Epigallocatechin Gallate. Thromb. Res..

[61] Marinovic M.P., Morandi A.C., Otton R. (2015). Green tea catechins alone or in combination alter functional parameters of human neutrophils via suppressing the activation of TLR-4/NFκB p65 signal pathway. Toxicol. In Vitro.

[62] Dryden G.W., Song M., Mcclain C. (2006). Polyphenols and gastrointestinal diseases. Curr. Opin. Gastroenterol..

[63] Kawai K., Tsuno N.H., Kitayama J., Okaji Y., Yazawa K., Asakage M., Sasaki S., Watanabe T., Takahashi K., Nagawa H. (2005). Epigallocatechin gallate induces apoptosis of monocytes. J. Allergy Clin. Immunol..

[64] Matsui T. (2015). Condensed catechins and their potential health-benefits. Eur. J. Pharmacol..

[65] Aura A.M., Mattila I., Seppänen-Laakso T., Miettinen J., Oksman-Caldentey K.M., Orešič M. (2008). Microbial metabolism of catechin stereoisomers by human faecal microbiota: Comparison of targeted analysis and a non-targeted metabolomics method. Phytochem. Lett..

[66] Clifford M.N., Van der Hooft J.J., Crozier A. (2013). Human studies on the absorption, distribution, metabolism, and excretion of tea polyphenols. Am. J. Clin. Nutr..

[67] Feng W.Y. (2006). Metabolism of Green Tea Catechins: An Overview. Curr. Drug Metab..

[68] Stalmach A., Mullen W., Steiling H., Williamson G., Lean M.E., Crozier A. (2010). Absorption, metabolism, and excretion of green tea flavan-3-ols in humans with an ileostomy. Mol. Nutr. Food Res..

[69] Chen Z., Zheng S., Li L., Jiang H. (2014). Metabolism of Flavonoids in Human: A Comprehensive Review. Curr. Drug Metab..

[70] Liu Y., Liu Y., Dai Y., Xun L., Hu M. (2003). Enteric Disposition and Recycling of Flavonoids and Ginkgo Flavonoids. J. Altern. Complement. Med..

[71] Bohn T., Mcdougall G.J., Alegría A., Alminger M., Arrigoni E., Aura A.M., Brito C., Cilla A., El S.N., Karakaya S. (2015). Mind the gap-deficits in our knowledge of aspects impacting the bioavailability of phytochemicals and their metabolites—A position paper focusing on carotenoids and polyphenols. Mol. Nutr. Food Res..

[72] Del R.D., Calani L., Cordero C., Salvatore S., Pellegrini N., Brighenti F. (2010). Bioavailability and catabolism of green tea flavan-3-ols in humans. Nutrition.

[73] Ottaviani J.I., Momma T.Y., Heiss C., Kwik-Uribe C., Schroeter H., Keen C.L. (2011). The sterochemical configuration of flavanols influences the level and metabolism of flavanols in humans and their biological activity in vivo. Free Radic. Biol. Med..

[74] Rio D.D., Rodriguezmateos A., Spencer J.P.E., Tognolini M., Borges G., Crozier A. (2013). Dietary (poly)phenolics in Human Health: Structures, Bioavailability, and Evidence of Protective Effects Against Chronic Diseases. Antioxid. Redox Signal..

[75] Neilson A.P., Sapper T.N., Janle E.M., Rudolph R., Matusheski N.V., Ferruzzi M.G. (2010). Chocolate Matrix Factors Modulate the Pharmacokinetic Behavior of Cocoa Flavan-3-OL Phase-II Metabolites Following Oral Consumption by Sprague-Dawley Rats. J. Agric. Food Chem..

[76] Chiou Y.S., Ma J.L., Sang S., Ho C.T., Wang Y.J., Pan M.H. (2012). Peracetylated (–)-Epigallocatechin-3-gallate (AcEGCG) Potently Suppresses Dextran Sulfate Sodium-Induced Colitis and Colon Tumorigenesis in Mice. J. Agric. Food Chem..

[77] Alzoghaibi M.A. (2013). Concepts of oxidative stress and antioxidant defense in Crohn’s disease. World J. Gastroenterol..

[78] Kruidenier L., Kuiper I., Lamers C.B., Verspaget H.W. (2003). Intestinal oxidative damage in inflammatory bowel disease: semi-quantification, localization, and association with mucosal antioxidants. J. Pathol..

[79] Forman H.J., Maiorino M., Ursini F. (2010). Signaling Functions of Reactive Oxygen Species. Biochemistry.

[80] Soobrattee M.A., Bahorun T., Aruoma O.I. (2006). Chemopreventive actions of polyphenolic compounds in cancer. BioFactors.

[81] Najafzadeh M., Reynolds P.D., Baumgartner A., Anderson D. (2009). Flavonoids inhibit the genotoxicity of hydrogen peroxide (H_2_O_2_) and of the food mutagen 2-amino-3-methylimadazo[4,5-*f*]-quinoline (IQ) in lymphocytes from patients with inflammatory bowel disease (IBD). Mutagenesis.

[82] Pereira R.B., Sousa C., Costa A., Andrade P.B., Valentão P. (2013). Glutathione and the Antioxidant Potential of Binary Mixtures with Flavonoids: Synergisms and Antagonisms. Molecules.

[83] Bors W., Heller W., Michel C., Saran M. (1990). Flavonoids as Antioxidants: Determination of Radical-Scavenging Efficiencies. Methods Enzymol..

[84] Kim C.Y., Lee C., Park G.H., Jang J.H. (2009). Neuroprotective Effect of Epigallocatechin-3-gallate against β-Amyloid-induced Oxidative and Nitrosative Cell Death via Augmentation of Antioxidant Defense Capacity. Arch. Pharm. Res..

[85] Bao G.H., Xu J., Hu F.L., Wan X.C., Deng S.X., Barasch J. (2013). EGCG inhibit chemical reactivity of iron through forming an Ngal-EGCG-iron complex. BioMetals.

[86] Raza H., John A. (2007). In vitro protection of reactive oxygen species-induced degradation of lipids, proteins and 2-deoxyribose by tea catechins. Food Chem. Toxicol..

[87] Guo Q., Zhao B., Li M., Shen S., Xin W. (1996). Studies on protective mechanisms of four components of green tea polyphenols against lipid peroxidation in synaptosomes. Biochim. Biophys. Acta.

[88] Meng Q., Velalar C.N., Ruan R. (2008). Effects of epigallocatechin-3-gallate on mitochondrial integrity and antioxidative enzyme activity in the aging process of human fibroblast. Free Radic. Biol. Med..

[89] Esworthy R.S., Aranda R., Martín M.G., Doroshow J.H., Binder S.W., Chu F.F. (2001). Mice with combined disruption of *Gpx1* and *Gpx2* genes have colitis. Am. J. Physiol. Gastrointest. Liver Physiol..

[90] Martín M.Á., Serrano A.B., Ramos S., Pulido M.I., Bravo L., Goya L. (2010). Cocoa flavonoids up-regulate antioxidant enzyme activity via the ERK1/2 pathway to protect against oxidative stress-induced apoptosis in HepG2 cells. J. Nutr. Biochem..

[91] Abboud P.A., Hake P.W., Burroughs T.J., Odoms K., O’Connor M., Mangeshkar P., Wong H.R., Zingarelli B. (2008). Therapeutic effect of epigallocatechin-3-gallate in a mouse model of colitis. Eur. J. Pharmacol..

[92] Seegert D., Rosenstiel P., Pfahler H., Pfefferkorn P., Nikolaus S., Schreiber S. (2001). Increased expression of IL-16 in inflammatory bowel disease. Gut.

[93] Wu X., Shao F., Yang Y., Gu L., Zheng W., Wu X., Gu Y., Shu Y., Sun Y., Xu Q. (2014). Epigallocatechin-3-gallate sensitizes IFN-γ-stimulated CD4^+^ T cells to apoptosis via alternative activation of STAT1. Int. Immunopharmacol..

[94] Kang K.S., Kang B.S., Lee B.J., Che J.H., Li G.X., Trosko J.E., Lee Y.S. (2002). Preventive effect of epicatechin and ginsenoside Rb_2_ on the inhibition of gap junctional intercellular communication by TPA and H_2_O_2_. Cancer Lett..

[95] Trosko J.E., Chang C.C., Upham B., Wilson M. (1998). Epigenetic toxicology as toxicant-induced changes in intracellular signalling leading to altered gap junctional intercellular communication. Toxicol. Lett..

[96] Kang N.J., Lee K.M., Kim J.H., Bo K.L., Kwon J.Y., Lee K.W., Lee H.J. (2008). Inhibition of Gap Junctional Intercellular Communication by the Green Tea Polyphenol (–)-Epigallocatechin Gallate in Normal Rat Liver Epithelial Cells. J. Agric. Food Chem..

[97] Sai K., Kanno J., Hasegawa R., Hasegawa R., Trosko J.E., Inoue T. (2000). Prevention of the down-regulation of gap junctional intercellular communication by green tea in the liver of mice fed pentachlorophenol. Carcinogenesis.

[98] Ale-Agha N., Stahl W., Sies H. (2002). (–)-Epicatechin effects in rat liver epithelial cells: Stimulation of gap junctional communication and counteraction of its loss due to the tumor promoter 12-*O*-tetradecanoylphorbol-13-acetate. Biochem Pharmacol..

[99] Sigler K., Ruch R.J. (1993). Enhancement of gap junctional intercellular communication in tumor promoter-treated cells by components of green tea. Cancer Lett..

[100] Lee S.J., Lee K.W., Lee H.J. (2004). Abies *nephrolepis* leaf phenolics prevent the inhibition of gap junction intercellular communication by hydrogen peroxide in rat liver epithelial cells. BioFactors.

[101] Zhao Y., Yu L., Xu S., Qiu F., Fan Y., Fu G. (2011). Down-regulation of connexin43 gap junction by serum deprivation in human endothelial cells was improved by (–)-epigallocatechin gallate via ERK MAP kinase pathway. Biochem. Biophys. Res. Commun..

[102] Yu L., Zhao Y., Fan Y., Wang M., Xu S., Fu G. (2010). Epigallocatechin-3 Gallate, a Green Tea Catechin, Attenuated the Downregulation of the Cardiac Gap Junction Induced by High Glucose in Neonatal Rat Cardiomyocytes. Cell. Physiol. Biochem..

[103] May M.J., Ghosh S. (1998). Signal transduction through NF-κB. Immunol. Today.

[104] Ukil A., Maity S., Das P.K. (2006). Protection from experimental colitis by theaflavin-3,3′-digallate correlates with inhibition of IKK and NF-κB activation. Br. J. Pharmacol..

[105] Andújar I., Recio M.C., Giner R.M., Cienfuegos-Jovellanos E., Laghi S., Muguerza B., Ríos J.L. (2011). Inhibition of Ulcerative Colitis in Mice after Oral Administration of a Polyphenol-Enriched Cocoa Extract Is Mediated by the Inhibition of STAT1 and STAT3 Phosphorylation in Colon Cells. J. Agric. Food Chem..

[106] Jung M., Triebel S., Anke T., Richling E., Erkel G. (2009). Influence of apple polyphenols on inflammatory gene expression. Mol. Nutr. Food Res..

[107] Khajah M.A., Fateel M.M., Ananthalakshmi K.V., Luqmani Y.A. (2016). Anti-Inflammatory Action of Angiotensin 1-7 in Experimental Colitis. PLoS ONE.

[108] Krens S.F.G., Spaink H.P., Snaar-Jagalska B.E. (2006). Functions of the MAPK family in vertebrate development. FEBS Lett..

[109] Huang S.M., Wu C.H., Yen G.C. (2006). Effects of flavonoids on the expression of the pro-inflammatory response in human monocytes induced by ligation of the receptor for AGEs. Mol. Nutr. Food Res..

[110] Lee J.H., Jin H., Shim H.E., Kim H.N., Ha H., Lee Z.H. (2010). Epigallocatechin-3-gallate Inhibits Osteoclastogenesis by Down-Regulating c-Fos Expression and Suppressing the Nuclear Factor-κB Signal. Mol. Pharmacol..

[111] Danesi F., Philpott M., Huebner C., Bordoni A., Ferguson L.R. (2010). Food-derived bioactives as potential regulators of the IL-12/IL-23 pathway implicated in inflammatory bowel diseases. Mutat. Res..

[112] Moon D.O., Choi S.R., Lee C.M., Kim G.Y., Lee H.J., Park Y.M. (2005). Epigallocatechin-3-gallate Suppresses Galactose-α1,4-galactose-β1,4-glucose Ceramide Expression in TNF-α Stimulated Human Intestinal Epithelial Cells Through Inhibition of MAPKs and NF-κB. J. Korean Med. Sci..

[113] Kensler T.W., Wakabayashi N. (2010). Nrf2: Friend or foe for chemoprevention?. Carcinogenesis.

[114] Bahia P.K., Rattray M., Williams R.J. (2008). Dietary flavonoid (–)-epicatechin stimulates phosphatidylinositol 3-kinase-dependent antioxidant response element activity and up-regulates glutathione in cortical astrocytes. J. Neurochem..

[115] Wakabayashi N., Slocum S.L., Skoko J.J., Shin S., Kensler T.W. (2010). When NRF2 Talks, who’s Listening?. Antioxid. Redox Signal..

[116] Liu G.H., Qu J., Shen X. (2008). NF-κB/p65 antagonizes Nrf2-ARE pathway by depriving CBP from Nrf2 and facilitating recruitment of HDAC3 to MafK. Biochim. Biophys. Acta.

[117] Schreiber S., Rosenstiel P., Hampe J., Nikolaus S., Groessner B., Schottelius A., Kühbacher T., Hämling J.J., Fölsch U.R., Seegert D. (2002). Activation of signal transducer and activator of transcription (STAT) 1 in human chronic inflammatory bowel disease. Gut.

[118] Sugimoto K. (2008). Role of STAT3 in inflammatory bowel disease. World J. Gastroenterol..

[119] Pandurangan A.K., Mohebali N., Esa N.M., Looi C.Y., Ismail S., Saadatdoust Z. (2015). Gallic acid suppresses inflammation in dextran sodium sulfate-induced colitis in mice: Possible mechanisms. Int. Immunopharmacol..

[120] Xue B., Xie J., Huang J., Chen L., Gao L., Ou S., Wang Y., Peng X. (2016). Plant Polyphenols Alter a Pathway of Energy Metabolism by Inhibiting Fecal Bacteroidetes and Firmicutes in vitro. Food Funct..

[121] Flint H.J., Scott K.P., Louis P., Duncan S.H. (2012). The role of the gut microbiota in nutrition and health. Nat. Rev. Gastroenterol. Hepatol..

[122] Pu S., Khazanehei H., Jones P.J., Khafipour E. (2016). Interactions between Obesity Status and Dietary Intake of Monounsaturated and Polyunsaturated Oils on Human Gut Microbiome Profiles in the Canola Oil Multicenter Intervention Trial (COMIT). Front. Microbiol..

[123] Walker A.W., Sanderson J.D., Churcher C., Parkes G.C., Hudspith B.N., Rayment N., Brostoff J., Parkhill J., Dougan G., Petrovska L. (2011). High-throughput clone library analysis of the mucosa-associated microbiota reveals dysbiosis and differences between inflamed and non-inflamed regions of the intestine in inflammatory bowel disease. BMC Microbiol..

[124] Frank D.N., St. Amand A.L., Feldman R.A., Boedeker E.C., Harpaz N., Pace N.R. (2007). Molecular-phylogenetic characterization of microbial community imbalances in human inflammatory bowel diseases. Proc. Natl. Acad. Sci. USA.

[125] Stevenson D.E., Hurst R.D. (2007). Polyphenolic phytochemicals—Just antioxidants or much more?. Cell. Mol. Life Sci..

[126] Archana S., Abraham J. (2011). Comparative analysis of antimicrobial activity of leaf extracts from fresh green tea and black tea on pathogens. J. Appl. Pharm. Sci..

[127] Rastmanesh R. (2011). High polyphenol, low probiotic diet for weight loss because of intestinal microbiota interaction. Chem. Biol. Interact..

[128] Inoue Y., Trevanich S., Tsujimoto Y., Miki T., Miyabe S., Sugiyama K., Izawa S., Kimura A. (1996). Evaluation of catechin and its derivatives as antioxidant: Recovery of growth arrest of Escherichia coli, under oxidative conditions. J. Sci. Food Agric..

[129] Ikigai H., Nakae T., Hara Y., Shimamura T. (1993). Bactericidal catechins damage the lipid bilayer. Biochim. Biophys. Acta.

[130] Kutschera M., Engst W., Blaut M., Braune A. (2011). Isolation of catechin-converting human intestinal bacteria. J. Appl. Microbiol..

[131] Beaud D., Tailliez P., Anbamondoloni J. (2005). Genetic characterization of the β-glucuronidase enzyme from a human intestinal bacterium, *Ruminococcus gnavus*. Microbiology.

[132] Jiménez-Girón A., Ibáñez C., Cifuentes A., Simó C., Muñoz-González I., Martín-Álvarez P.J., Bartolomé B., Moreno-Arribas M.V. (2015). Faecal metabolomic fingerprint after moderate consumption of red wine by healthy subjects. J. Proteome Res..

[133] Jin J.S., Lee K.C., Park I.S., Kim K.K., Ahn J.S., Benno Y., Hattori M., Lee J.S. (2014). *Gordonibacter faecihominis* sp. nov., isolated from human faeces. Antonie Leeuwenhoek..

[134] Jin J.S., Hattori M. (2012). Isolation and Characterization of a Human Intestinal Bacterium *Eggerthella* sp. CAT-1 Capable of Cleaving the C-Ring of (+)-Catechin and (–)-Epicatechin, Followed by *p*-Dehydroxylation of the B-Ring. Biol. Pharm. Bull..

[135] Wang L.Q., Meselhy M.R., Li Y., Nakamura N., Min B.S., Qin G.W., Hattori M. (2002). The Heterocyclic Ring Fission and Dehydroxylation of Catechins and Related Compounds by *Eubacterium* sp. Strain SDG-2, a Human Intestinal Bacterium. Chem. Pharm. Bull..

[136] Silverman R.B. (2002). The Organic Chemistry of Enzyme Catalyzed Reactions. Richard B. Silverman. The Organic Chemistry of Enzyme-Catalyzed Reactions.

[137] Du L., Wang Z., Zhao Y., Huang J., Pang H., Wei Y., Lin L., Huang R. (2014). A β-glucosidase from *Novosphingobium* sp. GX9 with high catalytic efficiency toward isoflavonoid glycoside hydrolysis and (+)-catechin transglycosylation. Appl. Microbiol. Biotechnol..

[138] Steed E., Balda M.S., Matter K. (2010). Dynamics and functions of tight junctions. Trends Cell Biol..

[139] Contreras T.C., Ricciardi E., Cremonini E., Oteiza P.I. (2015). (–)-Epicatechin in the prevention of tumor necrosis alpha-induced loss of Caco-2 cell barrier integrity. Arch. Biochem. Biophys..

[140] Salim S.Y., Söderholm J.D. (2011). Importance of Disrupted Intestinal Barrier in Inflammatory Bowel Diseases. Inflamm. Bowel Dis..

[141] Carrasco-Pozo C., Morales P., Gotteland M. (2013). Polyphenols Protect the Epithelial Barrier Function of Caco-2 Cells Exposed to Indomethacin through the Modulation of Occludin and Zonula Occludens-1 Expression. J. Agric. Food Chem..

